# Temperature-Regulated Synthesis of Hyaluronic Acid-Interpenetrated Polyacrylamide/Poly(Acrylic Acid Sodium Salt) Semi-Interpenetrated Polymer Network Gel for the Removal of Methyl Violet

**DOI:** 10.3390/gels10090556

**Published:** 2024-08-28

**Authors:** Nida Özcan, Nermin Orakdogen

**Affiliations:** 1 Department of Chemistry, Graduate School of Science Engineering and Technology, Istanbul Technical University, 34469 Istanbul, Turkey; 2 Soft Materials Research Laboratory, Department of Chemistry, Faculty of Science and Letters, Istanbul Technical University, 34469 Istanbul, Turkey

**Keywords:** semi-IPN, hyaluronic acid, swelling, elasticity

## Abstract

An alternative synthetic pathway was proposed for the optimization of synthesis to find a better correlation between the swelling and elasticity of hyaluronic acid-interpenetrated gels via temperature regulation. An experimental design methodology was presented for the synthesis of polyacrylamide/poly(acrylic acid sodium salt)/hyaluronic acid, PAAm/PSA/HyA, gels by modifying the one-pot procedure using free radical crosslinking copolymerization of AAm with the addition of anionic linear PSA chains in the presence of various amount of HyA, ranging between 0.05% and 0.20% (*w*/*v*). Semi-interpenetrated polymer network (IPN)-structured gels were designed with tunable elasticity, in which the extent of covalent crosslinking interactions is controlled by polymerization temperature ranging between −18 and 45 °C. Depending on the HyA content added in the synthesis and the polymerization temperature, the swelling ratio could be controlled. The addition of 0.05% (*w*/*v*) HyA increased the swelling of semi-IPNs, while the elastic modulus increased with increasing HyA content and decreased with the polymerization temperature. PAAm/PSA/HyA semi-IPNs showed the typical pH-sensitive swelling of anionic gels, and the swelling reached a maximum at a pH of 11.2. PAAm/PSA/HyA gels were tested for the removal of methyl violet from wastewater. Adsorption kinetics were shown to be well-fitted with the pseudo-second-order model using linear and nonlinear regression analysis. With the clear relationship between increased modulus and composition, this study enabled the fine-tuning of semi-IPN interactions by varying the polymerization temperature.

## 1. Introduction

Understanding the molecular-scale thermodynamic processes associated with polymerization temperature can provide new insights into the physical properties of polymer-based hybrid materials containing natural polysaccharides [1,2]. Glycosaminoglycans as the polymer backbone have provided a significant advantage in the hybrid design methodology that enables the one-pot synthesis of smart, switchable, or intelligent gels using negatively charged linear polysaccharides composed of repeating disaccharide units with multiple biological functions [3,4,5]. Designing dynamic hydrogels that can dissipate stress offers limited opportunity for in situ control because chemical modification is required to tune elasticity [6]. Host–guest molecular interactions provide a route to dynamically crosslink gels, producing dynamic materials using tunable elasticity on the bulk scale. These interactions can be tuned and modified by incorporating covalent crosslinking to some extent. This is usually achieved by incorporating linear polysaccharides with functional groups directly into the backbone or as side groups [7,8,9].

Hybrid gel synthesis is of interest with the use of linear polysaccharides, which impart various additional properties, such as biocompatibility, controlled structure, and elasticity, to the network structure with sufficient chemical functionality to bind guest molecules [10,11]. As a linear glycosaminoglycan, hyaluronic acid is composed of alternating repeating units of β-(1,4)-D-glucuronic acid and β-(1,3)-N-acetyl-D-glucosamine, which are linked by glycoside bonding [12,13]. As a weak polyacid, hyaluronic acid can be easily thiolated or methacrylated, producing hybrid networks with biologically relevant mechanics [14], and the mechanical properties, including elasticity and degradation resistance, can be modified using a variety of chemical reactions [15]. Hyaluronic acid is considered an important building block for the design of new materials that can be modified to alter the properties of polymer-based hybrid gels. In the chemical modification of hyaluronic acid, the carboxylic acid of glucuronic acid, primary and secondary hydroxyl groups, and the N-acetyl group play an active role through radical polymerization. Gel synthesis is achieved based on the creation of a radical that initiates the formation of a kinetic chain as a result of the reaction with the reactive group in the hyaluronic acid macromer. This provides various advantages, such as controlling the degree of reaction progress and being able to react in the presence of aqueous solutions. In addition to the known indisputable advantages of polyacrylamide (PAAm) gels, such as high hydrophilicity and biocompatibility, it is possible to improve some of their disadvantages, such as low strength and refractive index values, which limit their practical applications, by adding hyaluronidases.

The radically polymerized structures can be converted into functional materials with endless variations in shape and size in the presence of hyaluronic acid using various synthesis pathways. Atoufi and coworkers proposed a thermosensitive poly(N-isopropylacrylamide)/hyaluronic acid hydrogel synthesis procedure containing chitosan-g-acrylic acid coated micro/nanoparticles for cartilage tissue engineering [16]. Since unique design can be realized through material development, hydrogels with improved elasticity, low syneresis, and controlled drug release, as well as improved biocompatibility, have been prepared. The combined use of alkylacrylamide and hyaluronic acid increases the interrelationship between microscopic structure and mechanical–chemical properties, leading to tunability for the liquid/gel transition. Poly(alkylacrylamide) polymer is blended with hyaluronic acid to reduce syneresis and increase cellular binding. A recently developed way to improve the mechanical strength of PAAm hydrogels is to synthesize interpenetrating polymer networks (IPN) using hyaluronic acid–gelatin hydrogels and UV irradiation [17]. The gels are then swollen in acrylamide solution and crosslinked to prepare the IPN structure via UV irradiation. Thus, Yu and coworkers proposed a strategy to introduce PAAm as a flexible network to improve the elasticity of hydrogels with the IPN strategy. Kim and coworkers suggested another strategy to enhance pH sensitivity in poly(2-acrylamido-2-methylpropane sulfonic acid)/hyaluronic acid hydrogels via the sequential IPN method and provided a perspective, using hyaluronic acid as a pH-sensitive polymer, for the swelling over a wide pH range [18].

Covalent interpenetration of the gel components into macroscopic networks offers an alternative route for the radical polymerization of hyaluronic acid in the presence of cross-linkable counterparts. The covalent linkages between the hydrogel particles and the secondary hyaluronic acid matrix enable fibrillar structure formation around the particles. Based on the covalent interpenetration of microgels into macroscopic networks, Jha and coworkers reported the synthesis of hierarchical hyaluronic acid-based hydrogel matrices [19]. Compared with conventional bulk gels synthesized by photo-crosslinking hyaluronic acid, these hydrogels were both more flexible and fractured at higher strain while exhibiting a lower equilibrium swelling ratio. For the synthesis of hyaluronic acid-based core crosslinked nanogels, a simple method was proposed to adjust the crosslinking density. As a result of temperature-induced self-assembly, the formation of a hyaluronic acid-based nanogel was achieved via covalent crosslinking with the grafted copolymer chains and hydrazone bond formation [20]. Designing hybrid networks with hierarchical structures, mechanical strength, and the desired biological results is possible by both physically entrapping and covalently integrating hydrogel particles in a secondary hyaluronic acid-based network. In hyaluronic acid bulk gels, the interconnected network provides the macroscopic level, while the hydrogel particles limit the network to microscopic dimensions [20].

In the present work, a series of polyacrylamide/poly(acrylic acid sodium salt)/hyaluronic acid, PAAm/PSA/HyA, gels in semi-IPN structures were synthesized using free radical crosslinking polymerization by adjusting the content of AAm and PSA in the presence of various amount of hyaluronic acid (HyA). An alternative experimental procedure was proposed for the synthesis of alkylacrylamide-based gels, in which the polymerization temperature is varied with the aim of understanding the effect of anionic linear polymer PSA and hyaluronic acid addition on the physicochemical properties of semi-IPN gels. The main innovation of the proposed synthetic route is to perform the polymerization reaction at different temperatures between −18 °C and 45 °C, thus providing insight into the change in network structure depending on the synthesis temperature. The chemically modified polyacrylamide-based semi-IPN gels were synthesized to overcome their intrinsically non-anionic properties by incorporating linear PSA polymer-bearing carboxyl groups. As a result of dynamic interactions with linear PSA chains and the presence of different amounts of HyA, we aimed to provide molecular-level control over macroscopic material properties by linking them to the kinetics of polymerization at different temperatures. PAAm/PSA/HyA gels with four different HyA concentrations were synthesized via the sequential IPN method at four different polymerization temperatures. For this purpose, focusing on the synthesis of semi-IPN with low hyaluronic acid content between 0.05 and 0.20% (*w*/*v*), firstly the effect of the connection between the components on the physicochemical properties was investigated, and secondly, the selection of network architecture according to the analysis results and the relationship between the mechanical properties and the preparation temperature was evaluated. HyA content and polymerization temperature have been shown to be effective variables in adjusting the physico-mechanical properties of alkylacrylamide-based semi-IPN gels as desired. The properties of semi-IPN gels were fully characterized using Fourier transform infrared spectroscopy (ATR-FTIR), while the mechanical strength, dynamic, and equilibrium swelling profiles were studied as a function of the HyA content and preparation temperature. The controlled dye adsorption of semi-IPNs formed at different temperatures with varying HyA content was studied by employing methyl violet as a cationic dye. To construct and evaluate the adsorption process, the equilibrium adsorption data were fitted with various adsorption kinetic models. The method reported here will facilitate the design of novel nanostructured semi-IPNs with enhanced physicochemical properties depending on the polymerization conditions and improve the ability to predict and design their properties for future applications, enabling the synthesis of functional materials.

## 2. Results and Discussion

To understand the effect of linear polysaccharide content and polymerization temperature, the conventional synthesis method was modified by systematically changing these parameters while keeping all other parameters constant. The hypothesis of this study is to analyze the effect of both the addition of linear polysaccharides and the polymerization conducted at different temperatures on the network properties. By utilizing a combination of physical and covalent crosslinking in the HyA backbone, the gels with spatially controlled and tunable elasticity have been designed. The controllable mechanical properties of semi-IPN gels formed at different preparation temperatures were investigated, and the effect of increasing the pH of the external environment on the swelling of semi-IPNs in the basic region was studied. The relaxation of macromolecular chains observed as an overshoot effect on the macroscopic scale was explained in semi-IPNs formed at a higher polymerization temperature.

### 2.1. Structural Characterization of HyA-Containing Semi-IPN Gels

**Scheme 1:**
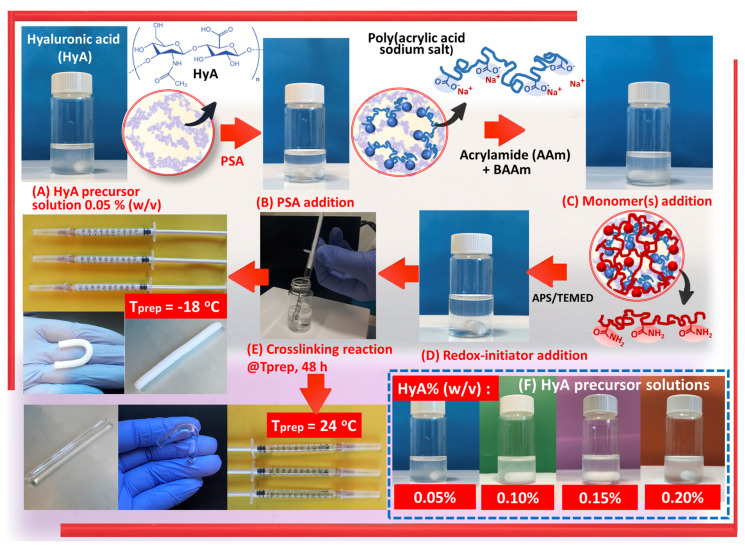
Synthesis route of semi-IPN PAAm/PSA/HyA gels. (**A**) Preparation of HyA precursor solution, (**B**) PSA addition, (**C**) monomer AAm and crosslinker BAAm addition, (**D**) redox-initiator pair APS/TEMED addition, (**E**) transfer to the polypropylene syringes and crosslinking reactions at different temperatures. (**F**) Appearances of HyA precursor solutions at different concentrations. Images of as-prepared-state semi-IPNs containing 0.05% (*w*/*v*) HyA after polymerization at −18 °C and 24 °C.

Semi-IPN gels of polyacrylamide/poly(acrylic acid sodium salt)/hyaluronic acid, PAAm/PSA/HyA, were synthesized using a one-pot procedure following the simultaneous free radical crosslinking copolymerization of nonionic monomer AAm, with the addition of anionic linear PSA chains (Scheme 1). Hyaluronic acid-free semi-IPN gels, PAAm/PSA, were prepared as a control group, and twenty different semi-IPN gels with anionic character were created, in which the nature and density of the structure were altered. The experimental design methodology was presented to optimize the synthesis parameters with a minimum number of experiments (Table 1). Factors affecting the semi-IPN preparation, such as polymerization temperature and network composition, were analyzed.

**Figure 1:**
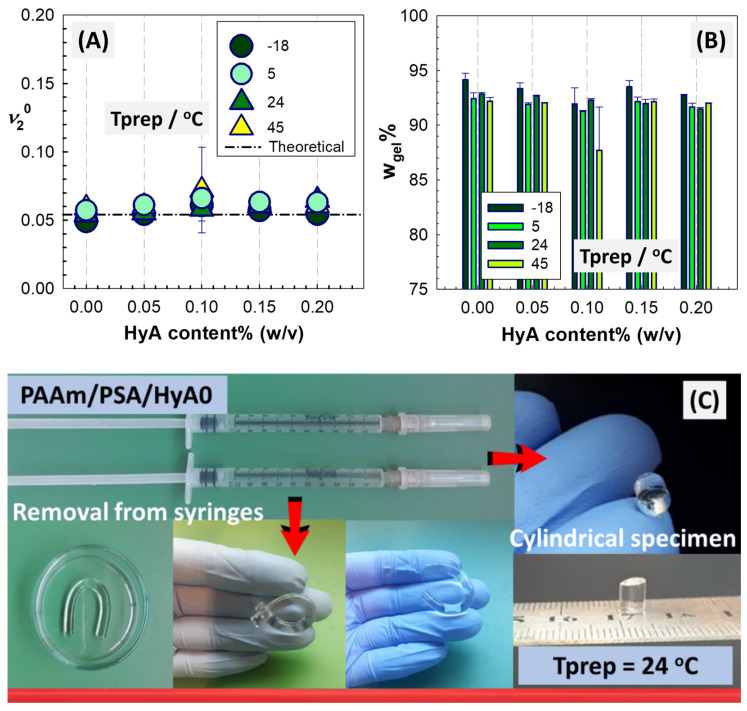
(**A**) Variation of experimental and theoretical values of volume fraction of crosslinked polymer network after preparation and (**B**) the gel fraction of PAAm/PSA/HyA semi−IPNs as a function of the polymerization temperature, as well as HyA content after complete drying. (**C**) Optical views of HyA−free control gel, PAAm/PSA/Hy0, synthesized at 24 °C.

Figure 1 shows the experimental and theoretical values $\nu_{2}^{0}$ of PAAm/PSA/HyA semi-IPNs as a function of the polymerization temperature and HyA content. The change in gel fraction with polymerization temperature and HyA content is also presented to evaluate the impact of AAm, BAAm, HyA, and PSA on the extent of crosslinking. It was observed that the gel fraction was affected by the polymerization temperature and the amount of HyA. The gel fraction shows the fraction of semi-IPN that is crosslinked due to the interactions among the monomer AAm, crosslinker BAAm, and linear polymers PSA and HyA during the polymerization process. The sol fraction remains un-crosslinked after the interpenetration of the different components and is not included in the network structure due to the absence of reactive sites during the polymerization depending on the polymerization temperature. The formation of the semi-IPN structure is initially based on the crosslinking of the AAm monomer in the presence of the crosslinker BAAm. The free radical mechanism to form the crosslinked PAAm structure is initiated by free radicals upon the addition of the catalyst TEMED in the presence of ammonium persulfate (APS). Due to the presence of the reactive double bond of AAm and the two reactive ends of BAAm that can engage in the crosslinking reactions between two growing chains and connect them together, a three-dimensional polymeric network is formed that traps the linear polymer chains [21]. PAAm, PSA, and HyA chains form the structure of the interpenetrated network gel due to the intermolecular polymer–polymer interactions. Depending on the increasing amount of HyA, the intermolecular interaction is determined by the dynamic mechanism of hydrogen bond formation and dissociation between the amide group of PAAm and the carboxylic acid group of the PSA and HyA chains.

Although there is a very small decrease in the gel fraction with increasing HyA amounts at a constant polymerization temperature, the gel fractions varying between 87 and 94% are high in all syntheses. In the PAAm/PSA/HyA formulation, as the HyA concentration increased from 0% to 0.20% (*w*/*v*) at 24 °C, the gel fraction varied between 92.8% and 91.4%, and relatively high gel fractions were obtained. With the increase in HyA content, the increase in carboxyl and hydroxyl groups in the network chains led to an increase in the reactive sites, resulting in an increase in the gel fraction. At a fixed HyA concentration, 0.10% HyA, the gel fraction decreased from 91.9 to 87.2% with the increase in polymerization temperature because HyA may degrade at an elevated temperature. Liaqat and coworkers prepared pH-responsive hyaluronic acid gels with methacrylic and acrylic acids via chemical crosslinking using BAAm and reached the same conclusions [22]. It has been reported that by increasing the carboxyl groups as reactive sites, the gel content increases with the increased number of reactive sites, resulting in greater contact between the polymer and monomers.

**Figure 2:**
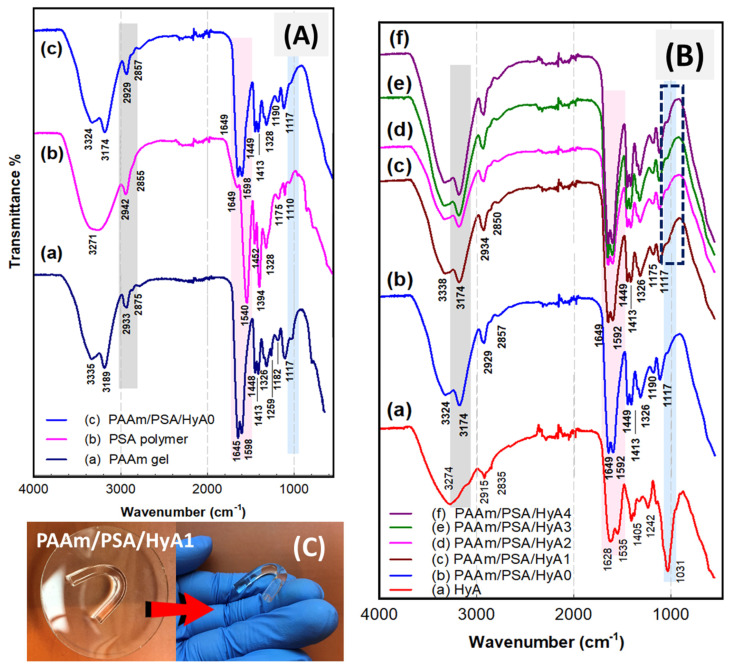
ATR−FTIR of (**A**) PAAm gel, linear PSA polymer, and HyA−free control gel, PAAm/PSA/HyA0, (**B**) neat hyaluronic acid (HyA) and PAAm/PSA/HyA semi−IPNs prepared with varying HyA contents. (**C**) As−prepared state appearance of 0.05% (*w*/*v*) HyA−containing PAAm/PSA/HyA1 semi−IPN sample synthesized at ambient temperature.

In Figure 2, the ATR-FTIR spectra of the HyA-free control gel, PAAm/PSA/Hy0, and HyA-containing PAAm/PSA/HyA semi-IPNs are depicted, and the attribution of the main bands are listed in Table S1. For structural comparison, the spectra of neat HyA, homopolymer PAAm gel, and linear PSA polymer were also included. As expected, the spectra of PAAm/PSA/HyA semi-IPNs are composed of bands from HyA and PAAm/PSA gel. Two bands centered at 1582 and 1625 cm^−1^ were ascribed to the N–H bending of amide II and C=O stretching of the carboxylate anion, respectively [23]. In the HyA spectrum, the region of 1200–950 cm^−1^ is characterized by the typical stretching of carbohydrates centered at 1150, 1077, 1044, and 948 cm^−1^. The ether bands were detected at 1150 and 948 cm^−1^ due to asymmetrical out-of-phase ring vibration. The C–O stretching vibration of carbinol resulting from the helical structure formed by hydrogen bonding within or between HyA molecules was detected at 1022 cm^−1^. The small peak at around 1024 cm^−1^ in PAAm/PSA/HyA, indicated by the dashed lines, confirmed the contribution of HyA, despite the inhibition of C–O elastic vibration as a result of crosslinking [24,25].

On the HyA spectrum, the bands observed at 2915 and 2853 cm^−1^ were assigned to the symmetric and asymmetric CH_2_ stretching, respectively. NH bending was detected at 1371 cm^−1^, and the symmetric stretching vibration of planar carboxyl groups C=O of HyA was observed at 1405 cm^−1^ [26]. For PAAm/PSA, the absorption at 3405–3410 cm^−1^ and 3190–3196 cm^−1^ was assigned to the asymmetric and symmetric stretching vibrations of the NH_2_ groups from AAm. The peak at 1413 cm^−1^ corresponds to the symmetric stretching of the carboxylate group of linear PSA, while the peak at 1379 cm^−1^ was assigned to the presence of N–H bonds related to -NH_2_ groups. At 1640–1649 cm^−1^, the carbonyl C=O stretching vibration (amide I) of amide groups of the AAm unit appeared in the PAAm/PSA and PAAm/PSA/HyA spectra. The band appearing at 1579 cm^−1^ was attributed to the bending vibration of the N–H of the primary amino -CONH_2_ groups. The N–H bending vibrations and the asymmetric stretching of the carboxylate group of PSA are superposed at 1579 cm^−1^ [27]. The peaks at 1160–1168 cm^−1^ are indicated C–C, while the peak belonging –CH_2_– bond formation appeared at 1455 cm^−1^. The asymmetric vibration of the C–H of CH_2_ groups was detected at around 2929–2931 cm^−1^ [28]. The band corresponding to the C–O–H deformation vibration and the stretching of the C–O bond was observed at 1286–1290 cm^−1^. The FTIR results show that the synthesis of the interpenetrating network hydrogel can successfully contain HyA, PAAm, and PSA polymer chains. A broad band centered at 3274 cm^−1^ was attributed to the O–H flex vibration of hydroxyl groups in HyA, while the shoulder seen at about 3105 cm^−1^ is due to NH stretching. The peaks of the N–H stretching of the amide group of PAAm and the O–H stretching of the carboxylic acid group of PSA overlap, forming the structure of the interpenetrating network hydrogel due to the intermolecular polymer–polymer interactions of PAAm and PSA. As seen, the OH band of the HyA-free control gel, PAAm/PSA, is of comparable intensity but narrower than that of the HyA-containing semi-IPN samples. The stretching vibrations of CN from the PAAm unit and the carboxylate of the PSA unit are superposed at around 1406 cm^−1^. The amide groups of PAAm serve as hydrogen bond acceptors, while the carboxylic acid groups serve as hydrogen bond donors. The intermolecular interaction is based on the dynamic mechanism of hydrogen bond formation and dissociation between the amide group of PAAm chains and the carboxylic acid group of HyA and PSA.

**Figure 3:**
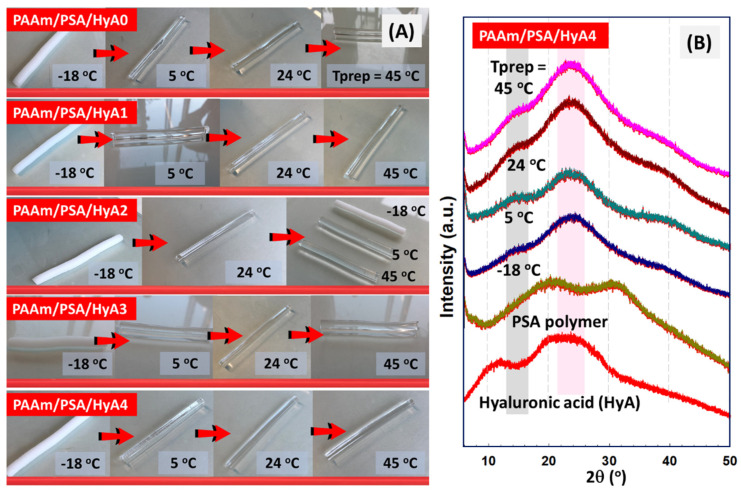
(**A**) As−prepared-state images of PAAm/PSA/HyA semi−IPNs synthesized at different polymerization temperatures after their removal from the syringe. (**B**) XRD curves of semi−IPNs as a function of the composition.

Figure 3B shows the X-ray diffraction patterns of raw HyA and semi-IPNs with various HyA content to analyze the polymer chain orientations in semi-IPNs. For the raw HyA pattern, the first peak at 11.83° was presented as sharper and lower-intensity. The data presented in the figure confirm that HyA is amorphous, with a broad peak ranging from 19.85 to 24.54°. The broadening of the diffraction peak is due to the interaction of amorphous areas with X-rays. For HyA, the XRD pattern was similar to that observed by Alcântara and coworkers for hyaluronic acid with a low molecular weight Mw (g/mol)~3.7 × 10^4^ g mol^−1^ [29]. These peaks suggest an ordered structure, in which the movement of the chains is hindered as a result of hydrogen bonds formed between the acetamido and hydroxyl groups [30]. The diffraction peaks observed at high 2θ values in the XRD pattern of HyA were attributed to the semi-crystalline nature of HyA due to the hydrogen bond interactions between the carboxylate, acetyl, and hydroxyl groups in the HyA chains [31]. After incorporation into the PAAm/PSA structure, these peaks disappear as a result of the formation of an amorphous structure in semi-IPNs. The diffractogram of the PAAm/PSA/HyA4 gel formed at 45 °C is characterized by an intense and broad signal at the angle of 2θ = 23.58° and a shoulder at 2θ = 37°, in accordance with other works [32]. A broad peak detected at 2θ = 23° indicated that a random network was formed by the interpenetration of PSA and HyA rather than a uniformly ordered network. The maximum peak was more pronounced at higher polymerization temperatures, while it was not so pronounced at lower temperatures due to the chain arrangement.

### 2.2. Swelling Properties of HyA-Containing Semi-IPN Gels

**Figure 4:**
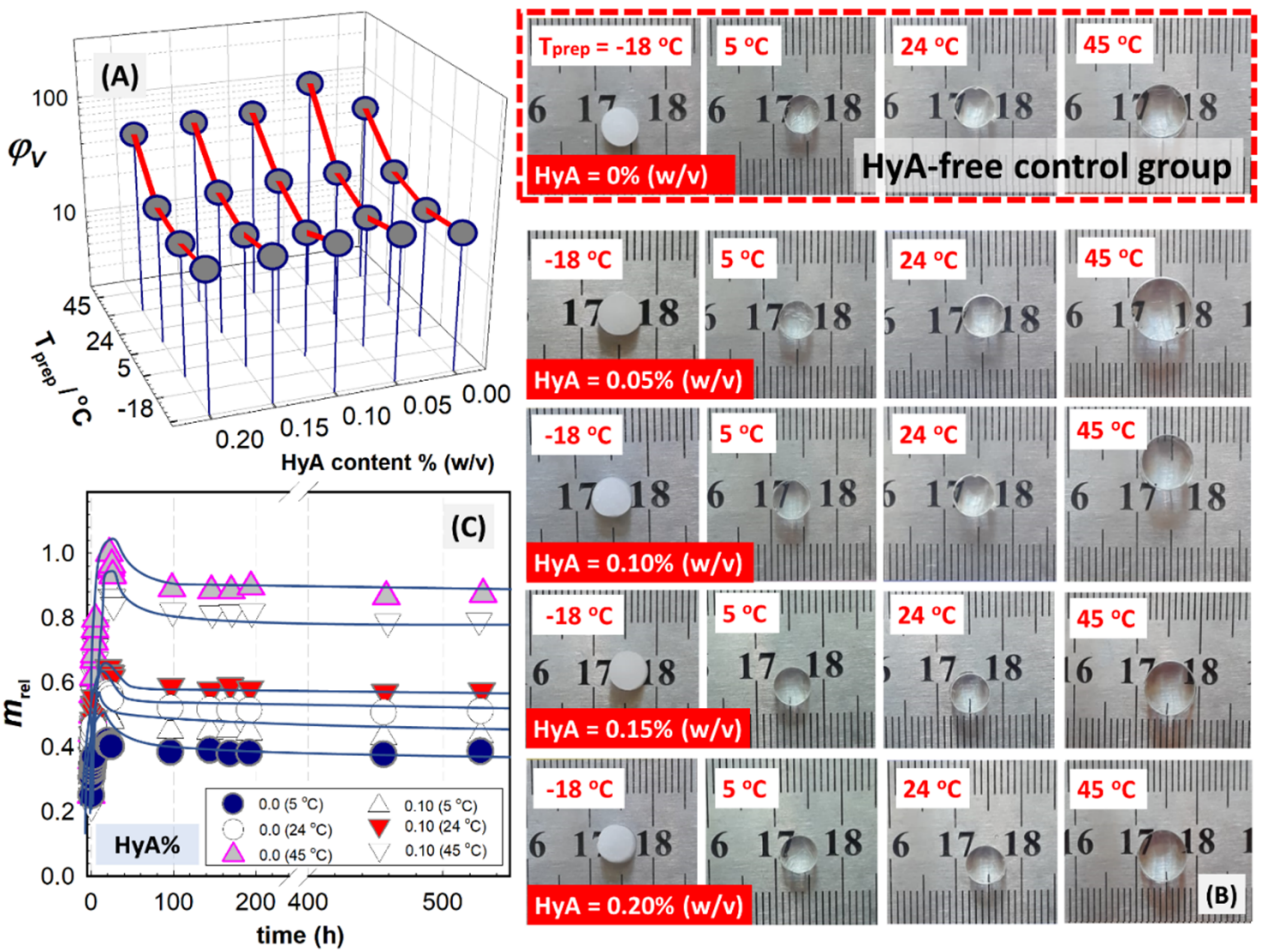
(**A**) Equilibrium volume swelling ratio of PAAm/PSA/HyA semi−IPNs shown as a function of HyA content, as well as the gel preparation temperature. (**B**) Optical images of semi−IPN gels after their swelling in water. (**C**) Swelling isotherms of PAAm/PSA/HyA semi−IPNs with different HyA content in terms of relative weight swelling ratio against the swelling time in water. The preparation temperatures were indicated.

**Figure 5:**
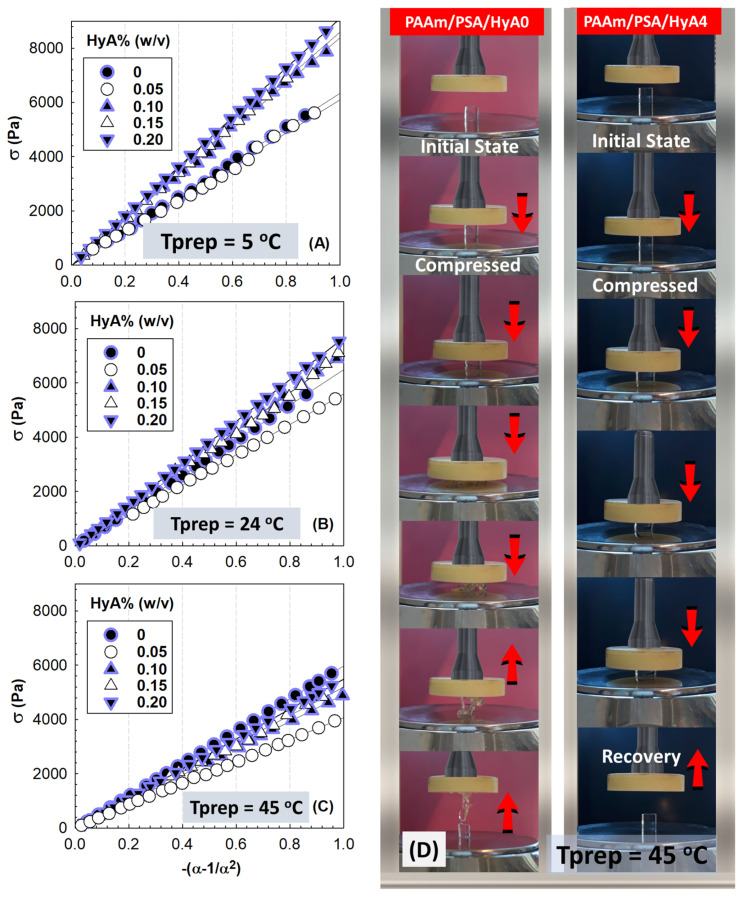
(**A**–**C**) Stress−compressive strain isotherms of PAAm/PSA/HyA semi−IPNs after their preparation at 5, 24, and 45 °C. HyA content. (**D**) Optical images of 0.20% (*w*/*v*) HyA−containing semi-IPN gels prepared at 45 °C during uniaxial compression testing.

In Figure 4, the equilibrium volume swelling ratio of PAAm/PSA/HyA semi-IPNs is shown as a function of HyA content, as well as the gel preparation temperature. The results were obtained with a large number of samples, including sixteen samples for HyA-containing PAAm/PSA/HyA gels and four samples for the HyA-free control group PAAm/PSA/HyA0, prepared under the same conditions and analyzed by following their swollen diameter. Both the polymerization temperature and the amount of HyA have an impact on the swelling capacity of semi-IPNs. Optical images of semi-IPN gels prepared at different temperatures after their swelling in water were presented. When a small amount of HyA, 0.05% (*w*/*v*), was added to the semi-IPN structure, the swelling first increased but then decreased with further increasing HyA content. The results indicated that high HyA concentrations may not be necessary to improve the swelling. Based on the behavior of semi-IPNs, the swelling results in Figure 4A are evaluated by dividing it into two areas. The first one is located between ambient temperature and −18 °C, while the second one is located beyond ambient temperature and at 45 °C. The swelling ratio of semi-IPNs formed at higher polymerization temperatures than ambient temperature is the highest, while the swelling ratio decreases as the temperature decreases. However, the swelling ratio of semi-IPN gels formed at temperatures below the freezing point of the polymerization solvent is higher than or close to ambient temperature conditions. Figure 5B presents the optical images of semi-IPN PAAm/PSA/HyA gels after their swelling in water. In addition to the effect of chemical composition on the swelling ability of gels due to the change in HyA content, their morphological structure change depending on the polymerization temperature. In the syntheses carried out at −18 °C, below the freezing point of water used as polymerization solvent, the PAAm/PSA/HyA gels appear opaque due to the formation of a porous structure as a result of cryogelation. However, PAAm/PSA/HyA gels synthesized at higher polymerization temperatures appear transparent, indicating the homogeneous network formation.

Ouasti and coworkers investigated the effect of network architecture on the elasticity and swelling of low-HyA semi-IPNs prepared using photopolymerized polyethylene diacrylate PEGDA [33]. The materials reported a higher probability of phase separation into HyA-rich and PEGDA-rich domains, indicating a lower swelling degree and correspondingly higher storage moduli ranging between 1440 and 2200 Pa. It was stated that since phase separation occurs during polymerization, macroscopic behavior is determined by more densely crosslinked and PEG-rich domains. Figure 3B shows the swelling kinetic curves of HyA-free control gel, PAAm/PSA gel, and semi-IPN PAAm/PSA/HyA2 gels containing 0.10%wt HyA prepared under different polymerization modes. In the figure, the dynamic swelling was presented against the swelling time in water. When analyzing the water uptake of the semi-IPN and control gels, an overshooting effect was observed. All semi-IPN gels showed a faster initial swelling rate and reached a maximum swelling and corresponding decrease in the swelling ratio until the equilibrium state. This phenomenon is expressed as the water uptake of semi-IPNs reaching a maximum in the early stages of swelling and then decreasing until the equilibrium state. The effect is observed more dominantly at increasing polymerization temperatures and HyA concentrations. The interpenetrating holes provide more storage space and shorter diffusion channels for the water molecules, thus favoring the swelling of semi-IPN PAAm/PSA/HyA gels. The overshooting phenomenon is related to the relaxation process of macromolecular chains. The water uptake reaches the equilibrium value as water is expelled from the network due to the elastodynamic forces of network chains. Díez-Peña et al. reported this phenomenon for the swelling of poly(N-isopropylacrylamide-co-methacrylic acid) semi-IPN gels, in which the overshooting effect was assigned to the formation of additional crosslinking [34]. The overshooting phenomenon occurs through the hydrogen bond formation between the carboxyl and amide groups of the network in a hydrophobic environment. Moreover, in lightly crosslinked gels, the overshooting phenomenon is explained by the lower crosslinking degrees and the relaxation of macromolecular chains. In semi-IPNs containing 0.1% (*w*/*v*) HyA formed at a higher polymerization temperature, a relaxation of macromolecular chains was detected, and this was observed as an overshoot effect on the macroscopic scale. Increased swelling allows for the rearrangement of the semi-IPN structure, which will also increase the mobility of the polymer chains [35]. In this way, the hydrogen bonds formed between the polymer chains containing AAm-, PSA-, and HyA-bearing carboxyl groups compete with the tendency of these units to form hydrogen bonds with water molecules. In addition, another reason for the overshooting effect is the presence of soluble polymer fractions in the gel that can be released into the solution after swelling.

### 2.3. Mechanical Properties of HyA-Containing Semi-IPN Gels

**Figure 6:**
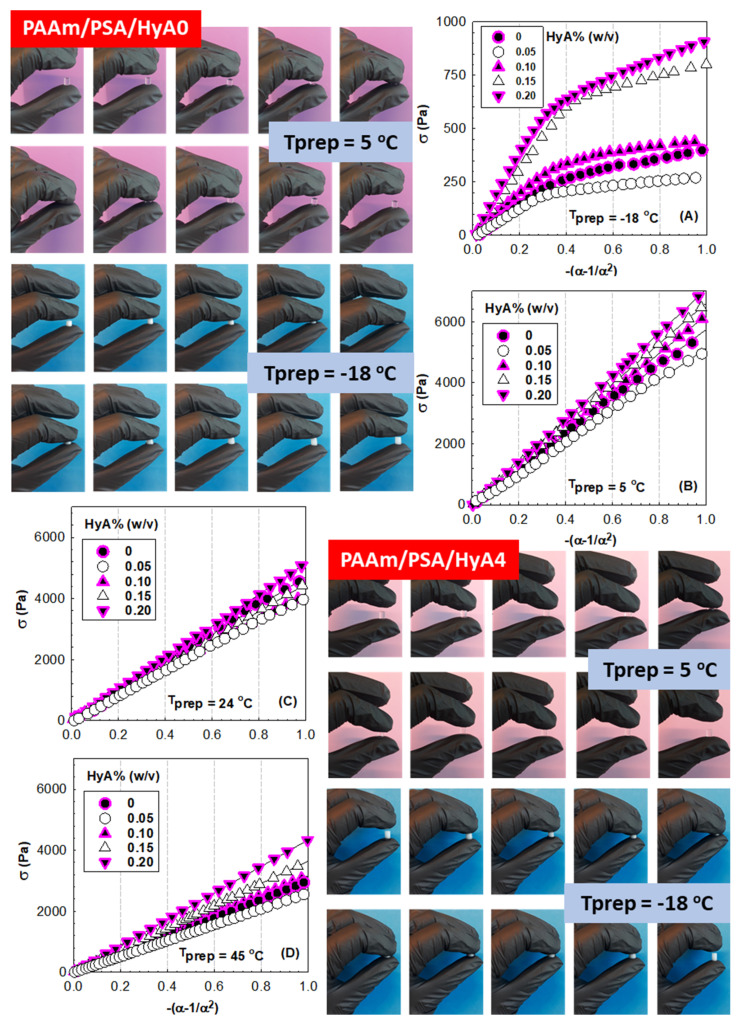
(**A**–**D**) Stress-compressive strain isotherms of semi-IPN PAAm/PSA/HyA gels after their equilibrium swelling in water. HyA content. Optical views of gels formed at different conditions after finger compression.

**Figure 7:**
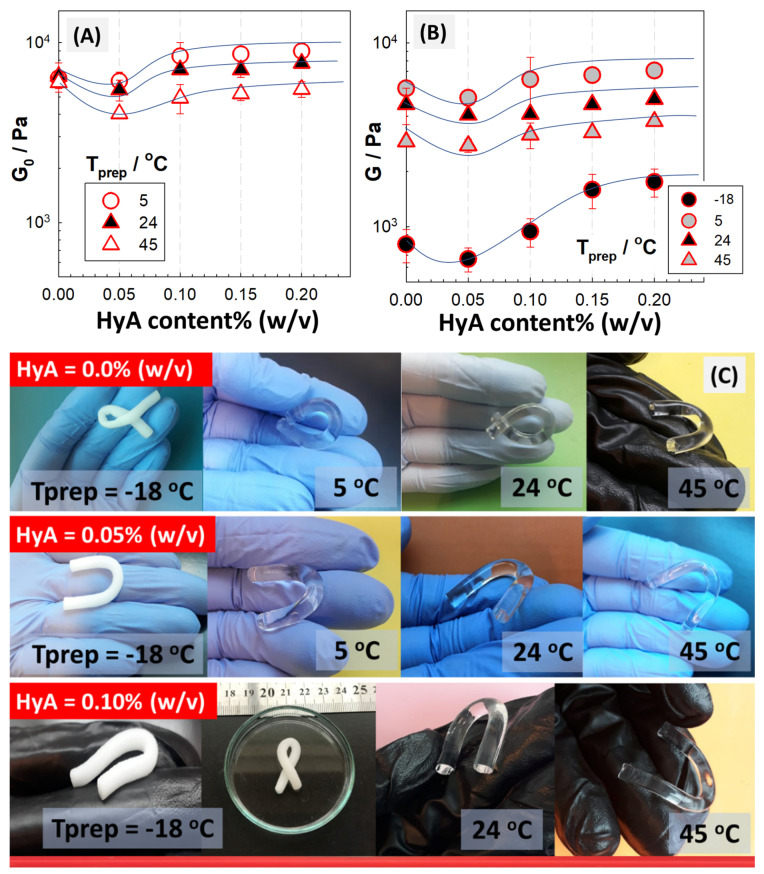
Compressive elastic moduli of PAAm/PSA/HyA gels from the uniaxial compression tests after preparation (**A**) and after equilibrium swelling in water (**B**) as a function of polymerization temperature. (**C**) Manual bending and folding images of semi-IPNs synthesized at different polymerization temperatures after removal from the syringe.

Figure 5 shows the stress-compressive strain isotherms of PAAm/PSA/HyA semi-IPNs after their preparation state at 5, 24, and 45 °C. While the after-synthesis moduli of semi-IPN gels formed at 5, 24, and 45 °C were measured, only the swollen modulus of gels formed at −18 °C was performed, since their network may contain frozen regions. Therefore, the stress-compressive strain isotherms of PAAm/PSA/HyA gels after their equilibrium swelling in water were collected in Figure 6. The slope of the curves changes depending on the polymerization temperature and the amount of HyA incorporated into the semi-IPNs. As the temperature increases, the slope of the curves decreases and becomes smaller at low deformation. After the swelling process in water, the compression strength becomes smaller. Figure 5D shows the optical images of 0.20% (*w*/*v*) HyA-containing semi-IPN gels formed at 45 °C during the uniaxial compression testing. While the HyA-free control gel, PAAm/PSA/HyA0, formed at a higher polymerization temperature without the addition of HyA moieties is broken due to its inability to dissipate the energy under 100% compression, semi-IPN gels containing 0.2% (*w*/*v*) HyA do not break as a result of the effective distribution of the compressive force by the presence of HyA chains. Except for semi-IPN cryogels prepared at −18 °C, all plots were linear over the stress range covered, and therefore, HyA-containing gels formed at temperatures of 5, 24, and 45 °C were assumed to be rubbery materials. The slope of each curve in Figure 5 and Figure 6 was analyzed using linear regression to determine the modulus of PAAm/PSA/HyA semi-IPNs. Figure 7 presents the variation of moduli of PAAm/PSA/HyA gels after preparation G_0_ and after equilibrium swelling in water G determined from the slope of the initial linear part, 3–5% of compressive strain isotherms. The optical views of the manual bending and folding of PAAm/PSA/HyA gels formed at different polymerization temperatures after removing them from the syringe were also presented in the figure. The compressive elastic modulus initially decreases with increasing HyA content and then tends to increase with further increases in the HyA content, which is evident in the swollen modulus values, indicating the increasing trend of elastic response of hydrogels. PAAm/PSA chains are flexible, providing flexibility to the network, while rigid hyaluronic acid chains increase mechanical strength. The modulus of semi-IPN gels changed according to the polymerization temperatures. As the swelling ratio of semi-IPNs increased with an increasing preparation temperature, lower modulus values were obtained. Figure S1 shows the crosslink density ν_e_ of PAAm/PSA/HyA gels formed at 5, 24, and 45 °C plotted against HyA%. Consistent with the higher swelling capacity of the semi-IPN gels formed at 45 °C, their moduli and crosslink density are lower than those formed at 5 °C, indicating that the crosslinking efficiency increases with decreasing the gelation temperature.

Similar results were reported for ethylene glycol diglycidyl ether crosslinked hyaluronic acid hydrogels prepared at 50 °C and 25 °C. It has been reported that increasing temperature decreases the crosslinking efficiency due to the partial degradation of HyA molecules producing shorter primary chains [36]. The hydrogels formed at 50 °C showed greater swelling in water compared to those prepared at 25 °C. Jeon and coworkers studied the elasticity of poly(ethylene glycol)-diamine crosslinked hyaluronic acid hydrogels prepared via covalent crosslinking at various crosslinking densities [37]. This study was conducted by controlling the elasticity of crosslinked hydrogels by varying both the crosslinking density and the molecular weight of crosslinking molecules. The modulus increased gradually as the crosslinking density of hydrogels increased from 0% to 20%, while a decrease in the modulus was observed as the crosslinking density increased above 20%. In another work, Pluda and coworkers analyzed the crosslinking parameters of HyA-based dermal fillers and showed that depending on the HyA concentration during crosslinking, alkaline conditions can affect the crosslinking efficiency, density, and hydrolysis kinetics [38].

### 2.4. pH-Response of HyA-Interpenetrated Semi-IPNs

**Figure 8:**
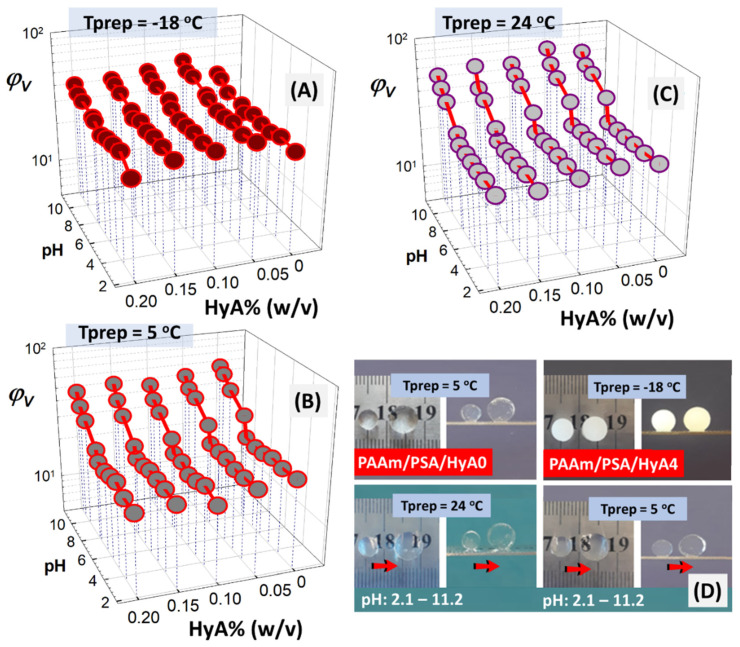
(**A**–**C**) Equilibrium volume swelling ratio of PAAm/PSA/HyA semi−IPN shown as a function of pH of swelling medium, as well as gel preparation temperature. (**D**) Optical images of semi−IPN PAAm/PSA/HyA after their swelling in acidic and basic pH conditions.

As the semi-IPN structure contains carboxyl groups in the backbone of HyA and PSA moieties, the swelling of semi-IPNs formed at different temperatures has been studied in media with different pH values (2.1–11.2) as a function of HyA content in Figure 8A–C, keeping in mind that pKa varies with the molecular weight and salt concentration and that the effective pKa of an acid group can vary by the extent of the interactions of hydrogen bonding. HyA is a weak polyanion with a pKa of 2.9, while the linear polyacid PSA is a weak polyanion with a pKa of approximately 4.72 [39,40]. The pH dependence of the degree of equilibrium swelling of HyA- and PSA-containing semi-IPNs showed a maximum at pH 11.2. While no significant increase in the swelling was observed in the pH range of 2.1–6.6, the swelling showed a rapid increase with increasing pH afterward. pH-sensitive swelling became more pronounced as the preparation temperature of semi-IPNs increased. pH-induced swelling for semi-IPNs prepared under low polymerization conditions, −18 °C, was not as pronounced as in high-temperature syntheses. This may be due to the low incorporation of PSA units into the structure under cryoconditions. Since the number of charges at a given pH will vary depending on the presence of other charged species, such as co-ions and counter-ions, the swelling will vary depending on the semi-IPN composition.

Mafe and coworkers reported that a different situation occurs, in which hydrogen bonding is more likely between the carboxylic acid groups than between the –COOH and –COO^−^ groups. When the solution contains enough salt cations to compensate for the ionized acid groups, hydrogen bonding between two neutral groups can alter the swelling equilibrium of the gel. As a result of this interaction, it has been stated that the apparent pKa increases because of the effective dissociation of acrylic acid in the gel, which is significantly reduced compared to the value without hydrogen bonding [41]. Laguecir and coworkers performed Monte Carlo coarse-grained simulations to analyze the role of pH on the ionization and conformation of poly(acrylic acid) PAA chains. The authors investigated PAA diffusion properties in aqueous solutions as a function of pH [39]. While high- and low-molecular weight PAA does not exhibit similar behavior, higher-molecular weight chains require more charge than lower-molecular weight chains to achieve significant elongation. This result is due to the direct induction of spatial extension by increasing the persistence length due to electrostatic repulsions resulting from ionization. Comparing the swelling for a certain HyA content, the swelling of semi-IPNs formed at 24 °C increased by 2.3 times when the swelling pH was increased from 2.1 to 11.2 by adding 0.20% HyA to the PAAm/PSA structure. Since the deprotonation of the carboxyl group -COOH within the network with increasing external pH leads to anionic carboxylate -COO^−^, the electrostatic repulsion between the negatively charged carboxylate anions causes the network to expand significantly. Mahon and coworkers reported similar results for pH-dependent swelling of sodium polyacrylate and poly(acrylamide-co-acrylic acid) potassium salt [42]. The swelling behavior of hydrogels typically shows maximum swelling between pH 6.5 and 9. This phenomenological change is attributed to the abundance of –COO^−^ groups that reduce the hydrogen bond interactions. For the present semi-IPNs, when the swelling at a certain pH value, pH 11.2, was compared, the swelling of semi-IPNs formed at 24 °C decreased by 15.8% when 0.20% HyA was added to the PAAm/PSA structure. In semi-IPNs formed at 5 °C, a decrease of 16.5% occurred when the same amount of HyA was added, which was proportional to the swelling trend in water. Kim and coworkers reported the effect of pH on the swelling property of hyaluronic acid-based hydrogel beads. After raising the pH from 4 to 6, the swelling increased dramatically by the presence of the carboxyl functional group in the side chains [43]. The highest swelling ratio was observed around pH 7~8, while the swelling decreased with increasing pH up to 10. The decrease in the swelling ratio of microbeads below pH 6 (acidic) and above pH 10 (basic) is attributed to the lack of ionization associated with the carboxyl groups.

### 2.5. Adsorption Properties of HyA-Interpenetrated Semi-IPN Gels

**Figure 9:**
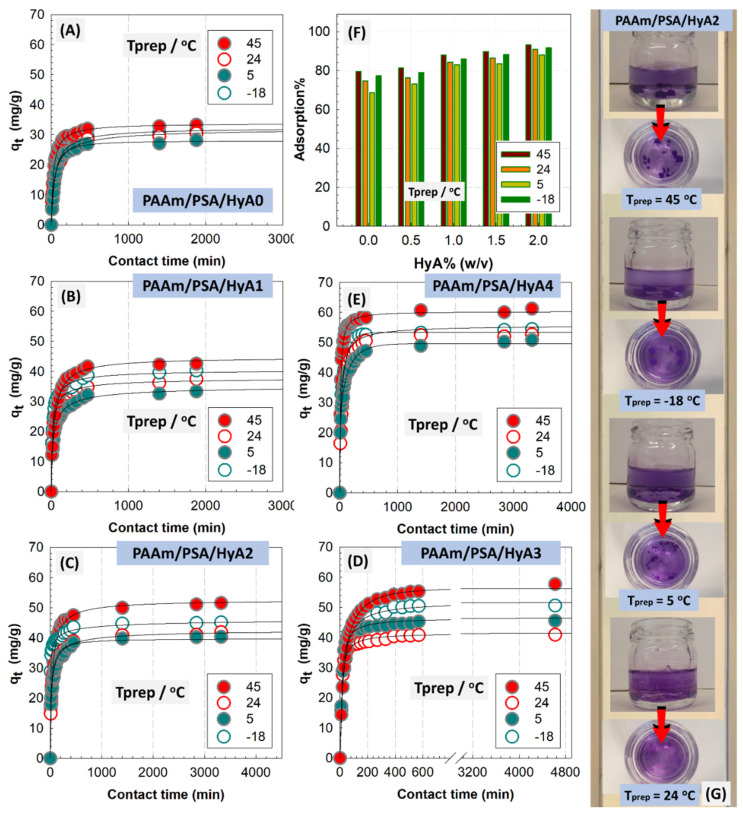
The adsorption performance (**A**–**E**), and adsorption capacity (**F**) of HyA-integrated PAAm/PSA/HyA gels as a function of contact time at different preparation conditions. (**G**) Optical images of HyA-integrated PAAm/PSA/HyA gels after 1 h adsorption of MV dye.

PAAm/PSA/HyA semi-IPNs were studied as adsorbents for the removal of cationic methyl violet dye since the structure of semi-IPNs contained amide and carboxylate functional groups that might be important for the process. The effect of the incorporated HyA on the adsorption efficiency of semi-IPNs was performed in batch mode under vigorous stirring. Figure 9 shows the adsorption percentage and capacity of semi-IPNs formed at different polymerization temperatures with varying HyA loadings as a function of the contact time. The optical images of HyA-integrated PAAm/PSA/HyA gels were presented in the figure (Figure S2). As observed, the removal efficiency is significantly increased with increasing HyA content in semi-IPNs. The HyA-free control gel was not able to effectively remove the MV dye, and the adsorption percentage ranged between 68.6 and 79.5%, depending on the polymerization temperature, while for semi-IPN containing 0.20% HyA, the efficiency reached 88.1–93.2%. The adsorption of MV on all semi-IPNs was rapid in the first 1 h, then gradually increased until reaching a plateau. This gradual increase was more pronounced in gels with low HyA content, while the adsorption rate increased as HyA was added to the structure. The rapid first stage involves the adsorption of easily adsorbed dye molecules onto accessible active sites by internal diffusion before all adsorbent sites are saturated. Figure S3 presents the adsorption of HyA-integrated PAAm/PSA/HyA gels as a function of different preparation temperatures. The decrease observed in the adsorption of semi-IPNs synthesized at 5 °C was due to a decrease in the polymerization temperature below ambient temperature, and more crosslinking points were formed in the gel structure via hydrogen bonding, which increased the crosslink density and thus decreased the swelling/adsorption capacity. Increasing the amount of HyA from 0% to 0.15% (*w*/*v*) in PAAm/PSA/HyAx semi-IPN gels formed at 45 °C improved the removal percentage of MV and the adsorption capacity at equilibrium increased from 34.4 to 61.1 mg/g. In this study, pseudo-first-order (PFO), pseudo-second-order (PSO), Avrami, fractional power, Elovich, and intraparticle diffusion model were used to analyze the experimental data according to Equations (1)–(4), respectively [44], as follows:

(1) $$\ln(q_{e}-q_{t})=\ln q_{e}-k_{1}t$$

(2) $$\frac{t}{q_{t}}=\frac{t}{q_{e}}+\frac{1}{k_{2}q_{e}^{2}}$$

(3) $$\ln\left[\ln\left(\frac{q_{e}}{q_{e}-q_{t}}\right)\right]=n_{Av}\ln k_{Av}+n_{Av}\ln t$$

(4) $$q_{t}=k_{diff}t^{1/2}+C$$

where *q_t_* and *q_e_* are the adsorption capacity at time *t* and at the equilibrium (mg g^−1^), respectively, *k*_1_ (min^−1^) and *k*_2_ (g (mg min)^−1^) are the constants of adsorption rates (Table S2).

**Figure 10:**
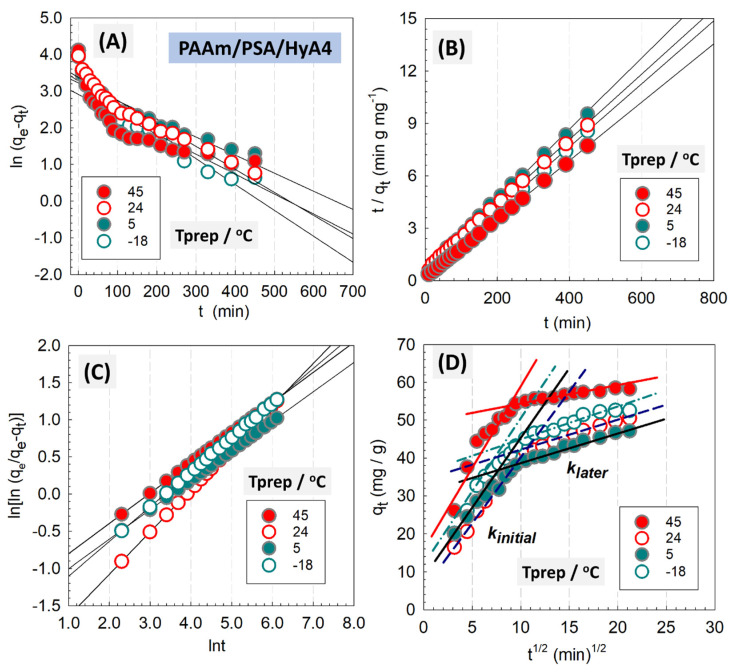
Regression analysis of adsorption of MV with 0.2% (*w*/*v*) HyA-integrated PAAm/PSA/HyA4 gels by pseudo-first-order (PFO) kinetic model (**A**), pseudo-second-order (PSO) kinetic model (**B**), Avrami model (**C**), and intraparticle diffusion model (**D**).

**Figure 11:**
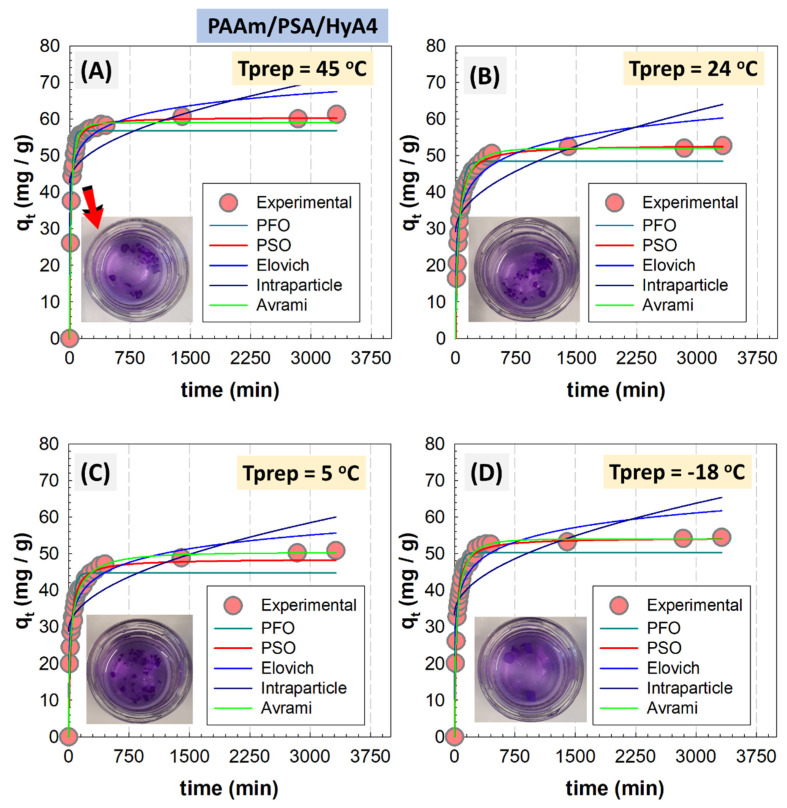
Results of nonlinear PFO kinetic model, PSO kinetic model, Avrami, Elovich kinetic model, and intraparticle diffusion model of 0.20% (*w*/*v*) PAAm/PSA/HyA4 semi−IPN gels formed at polymerization temperatures of Tprep (°C): 45 (**A**), 24 (**B**), 5 (**C**) and −18 (**D**).

From the syntheses of five different HyA compositions and four different polymerization temperatures, the semi-IPNs containing 0.1% and 0.2% (*w*/*v*) HyA were selected as models, and the analysis results of these gels are presented in Figure 10, Figures S4 and S5, respectively. The correlation coefficients obtained from the linear analysis of experimental data were listed in Table 2 and Tables S3–S6. Using Equation (1), the values *k*_1_ and *q_e_* were determined from the slope and intercept of the plot of $\ln(q_{e}-q_{t})$ against *t* in Figure 10A. In the same way, *k*_2_ and *q_e_* were calculated based on Equation (2) with graph *t*/*q_t_* against *t* in Figure 10B. As can be seen in Figure 10B and Figure S4, the PSO model for semi-IPNs showed a better fit of the experimental data as revealed by the R^2^ values (>0.99). The rather high values of R^2^ coefficients presented in Table 3 as well as the very close values of *q_e,calc_* to *q_e,exp_* indicated that the PSO model fits the experimental data better than all other models studied and provides evidence that the adsorption of MV onto semi-IPNs follows PSO kinetic model. This result indicates that MV adsorption is controlled by electron sharing or exchange between the solute and adsorbent. By applying nonlinear kinetic models, similar results were also found in Figure 11. Bakhshi and Darvishi studied the crystal violet adsorption onto hydrogel composites of starch-g-PNaMA/eggshell particles and reported that the dye adsorption followed the pseudo-second-order kinetics, indicating the rate-limiting step [45].

Using Equation (3), the linearized Avrami kinetic model was tested in Figure 10C as a double logarithmic form to determine Avrami kinetic constant *k*_Av_ and Avrami exponent *n*_Av_ of the time related to the change in the mechanism of adsorption. The Avrami model implies that the adsorption is located on the active surface sites of semi-IPNs, and the model can be used in cases in which the adsorption is slow and/or when there is more than one mechanism of adsorption [46]. This model assumes that the dyeing process covers a limited surface reaction, and the diffusion of dye molecules is rapid (*n_Av_* > 1). The Avrami model time exponent values associated with the adsorption mechanism varied in the range of 0.3918 < *n_Av_* < 0.5611, showing that the adsorption mechanism follows multiple kinetic patterns that change as a result of the contact of MV dye with semi-IPNs. Using Equation (4), the adsorbate pore diffusion activities were investigated using the intraparticle diffusion model, in which *k_diff_* is the intraparticle diffusion rate constant (mg g^−1^ min^−1/2^) and *C* is a constant that indicates the thickness of the boundary layer. MV adsorption onto semi-IPNs is governed by two steps, including intraparticle diffusion, as shown by the linear fit in Figure 10D and Figure S4D. Since the graph does not pass through the origin, intraparticle diffusion is not the only step for the adsorption mechanism of MV and is followed by a series of steps. The modeling parameters according to the linearized intraparticle diffusion model are presented in Table 2 for 0.20% HyA-containing PAAm/PSA/HyA4 semi-IPN gels, while the results were collected in Table S3 for 0.10% HyA-containing gels.

As observed in the nonlinear fitting of this model in Figure 11 and Figure S6, the values of the boundary layer thickness *C* were greater than zero. In addition, the higher *k_initial_* values observed in PAAm/PSA/HyA2 and PAAm/PSA/HyA4 gels reflected the enhanced porosity and strong affinity to MV dye for the prepared semi-IPNs. A similar observation was reported by Benhalima and coworkers for the removal of MV using Algerian diatomite-loaded hybrid biocomposites within hydrogel beads of a semi-interpenetrating network of carboxymethyl cellulose/dextran sulfate [47]. The Elovich model is effectively used to analyze the chemisorption process to explain the second-order kinetics with the assumption that solid surfaces are energetically heterogeneous. The model can be expressed in the following form:

(5) $$q_{t}=\frac{1}{\beta }\ln t+\frac{1}{\beta }\ln(\alpha \beta )$$

(6) $$\ln q_{t}=\ln k_{fr}+\nu_{fr}\ln t$$

where α (mg g^−1^ min^−1^) is the initial adsorption rate, and the *β* (g mg^−1^) is related to the extent of surface coverage defining the energy for chemisorption. Figure S5A,B shows the results for the Elovich model for semi-IPNs. According to the linear fit results in Table 2, the low R^2^ values ranging from 0.8666 to 0.9761 and the large deviation observed in the nonlinear fitting to the experimental results in Figure 11 showed that the adsorption could not be explained using the Elovich model. Using Equation (6), the fractional model was then tested to determine the fractional kinetic constants *k_fr_* and *ν_fr_* from the plots presented in Figure S5C,D. The fractional kinetic model relates the macroscopic properties to the adsorption dynamics depending on the residence times. The value of *ν_fr_* less than 1 shows that the adsorption kinetic data fit the fractional power model. Tawfik and Eltabey stated that the fractional kinetic model provides a physical meaning by examining the effect of retention times during adsorption processes [48].

**Scheme 2:**
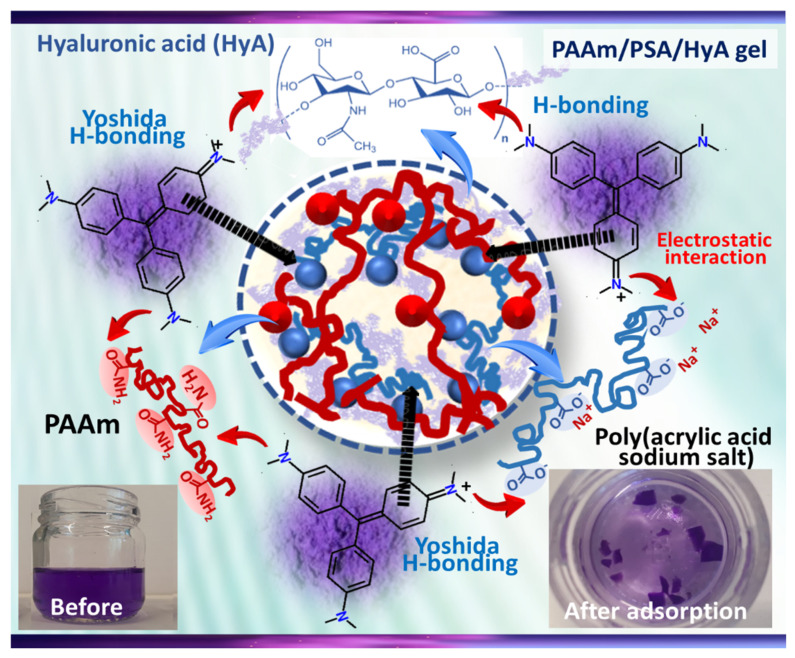
Illustration of the possible interactions between MV dye and HyA-containing PAAm/PSA/HyA semi-IPNs.

For the adsorption of MV onto semi-IPNs, the free energy change Δ*G*^0^ was evaluated using the van’t Hoff equation, $\Delta G^{0}=-RT\ln K_{C}$, in which the term *K*_c_ is the thermodynamic equilibrium constant of adsorption (L g^−1^), *T* is the absolute temperature in K, and *R* is the gas constant (8.314 J K^−1^mol^−1^). In the results presented in Table 3 and Table S6, the negative values of Δ*G*^0^ indicated the spontaneous and thermodynamically favorable adsorption of MV onto semi-IPNs. Meanwhile, the values of Δ*G*^0^ became more negative with increasing the polymerization temperature, indicating that higher temperature contributed to the adsorption (Figure S7). Rahchamani and coworkers studied the adsorption of MV from an aqueous solution by polyacrylamide and reported that the adsorption followed pseudo-second-order rate kinetics. From kinetic studies, the calculation for the enthalpy, free energy, and entropy changes showed that the MV–PAAm adsorption was favored at high temperatures [49]. Scheme 2 shows the illustration of the possible interactions between MV dye and HyA-containing PAAm/PSA/HyA semi-IPNs. In the structure of PAAm/PSA/HyA, the PAAm units contains large numbers of amide side groups and HyA backbone contains -NH and hydroxyl groups capable of entering into H-bond formation with -N=N- groups in MV molecules. The interaction between the positive charge on the tertiary nitrogen of MV dye and the negative charge of carbonyl groups of semi-IPN is the ion–ion interaction, the strong interchain connections, and hydrogen bonding [50]. Therefore, the suggested mechanism for MO adsorption involved H-bonding and pore-filling, as well as delocalized π-electron-specific interactions between the semi-IPN surface and the free electrons of the MV molecules present in the aromatic rings and multiple bonds.

## 3. Conclusions

An attempt was made to correlate the polymerization temperature, as well as linear polymer HyA content with the elasticity and swelling properties of semi-IPN PAAm/PSA/HyA gels. By conducting the free radical crosslinking of AAm, linear PSA, and HyA in water at a fixed crosslinker concentration, the semi-IPN gels were prepared at various gel preparation temperatures ranging between −18 and 45 °C. Synthetic routes performed to determine how the HyA content in the network and the polymerization temperature affect semi-IPN properties showed that the reaction at 45 °C and the structure containing 0.05 wt% HyA in the feed were beneficial in the formation of abundant interpenetrated structure in PAAm/PSA/HyA gels. Semi-IPNs formed at lower and higher polymerization temperatures compared to ambient temperature showed higher swelling. While the swelling was inversely proportional to the amount of HyA present, the ability of semi-IPNs to absorb water decreased with increasing HyA content in the network. HyA at a concentration of 0.05% (*w*/*v*) increased the swelling of semi-IPNs, while a further increase in HyA content negatively affected the swelling. In semi-IPNs prepared containing 0.1% (*w*/*v*) HyA at high polymerization temperature, a relaxation of the macromolecular chains was detected, indicating an overshooting effect at the macroscopic scale. The mechanical properties of crosslinked semi-IPNs were controlled by varying the HyA content and polymerization temperature. The elastic modulus increased by increasing HyA, while it decreased with temperature. pH-dependent swelling results showed that the low swelling ratio in the pH 2–6 range increased significantly in the basic pH region and reached a maximum at pH 11.2. When the potential use of PAAm/PSA/HyA semi-IPNs to adsorb MV dye was examined, the removal percentage of MV was improved by increasing the HyA content from 0.05% to 0.20% (*w*/*v*). The equilibrium adsorption data are well represented by pseudo-second-order model and the dye adsorption process was followed by a combined mechanism. Synthetic procedures that determine the effect of composition and polymerization temperature in the development of responsive materials with this interpenetrated network system were proposed and further research on micromorphology is required. This study provides accurate and effective methods, making a valuable contribution to the field of HyA-containing semi-IPN structured gel synthesis, depending on the polymerization temperature. In conclusion, this modular design approach to hydrogel production is anticipated to have applications in the removal of cationic dyes from wastewater and in pH-sensitive controlled release systems.

## 4. Experimental

### 4.1. Materials and Methods

Acrylamide (AAm) and poly(acrylic acid sodium salt) (PSA) with a weight average molecular weight of 5100 g mol^−1^ were purchased from Merck. Hyaluronic acid (HyA) from Rooster comb was generously donated by Bugamed Biotechnology, Turkey. The average molecular weight of HyA was determined by GPC using water as Mw (g/mol)~2.8 × 10^6^ g mol^−1^ % with Mw/Mn 1.361 (±7.222%). The crosslinker N,N-methylenebisacrylamide (BAAm), the redox-initiator system, ammonium persulfate (APS), and N,N,N′,N′-tetramethylethylenediamine (TEMED) were supplied from Merck and used as received. Disodium hydrogen phosphate (Merck), hydrochloric acid (Merck), potassium dihydrogen phosphate (Riedel-de Haen), and potassium phosphate (J.T. Baker) were used for pH-dependent swelling testing.

### 4.2. Synthetic Pathway for Preparation of Hyaluronic Acid-Interpenetrated Semi-IPN Gels

Semi-IPN gels of polyacrylamide/poly(acrylic acid sodium salt)/hyaluronic acid, PAAm/PSA/HyA, were synthesized using the one-pot procedure based on the simultaneous free radical crosslinking copolymerization of nonionic monomer AAm by addition of anionic linear PSA chains in the presence of various amount of HyA. The concentration of AAm and PSA was set to 7.1%wt and 1.5%wt, whereas the HyA concentration was changed between 0.05 and 0.20% (*w*/*v*) (Table 1). The crosslinker ratio *X*, the mole ratio of the crosslinker BAAm to the AAm, PSA, and HyA was fixed at 1/80. The desired amount of HyA was added to 7.0 mL of distilled water and left in the refrigerator overnight to swell. After mixing for 30 min, PSA (0.150 g), AAm (0.7108 g), and 1.0 mL of BAAm stock solution (0.388 g/20 mL water) were added to HyA precursor solution at 1 h intervals (A–C in Scheme 1) and dissolved at room temperature for 3 h with continuous stirring. Since AAm and PSA are solid, the mixing was performed carefully to avoid agglomeration after each addition. As seen in Scheme 1, a homogeneous solution was obtained after stirring for 1 h after each addition in steps (A)–(D). To initiate the polymerization reaction, 1.0 mL of APS stock solution (0.8 g APS/10 mL water) was added in stage (D) and continuously stirred for an additional 30 min at room temperature. After the addition of 1.0 mL of TEMED stock solution (750 μL/20 mL water), the solution was injected into several polypropylene syringes in stage (E) with a 4–5 mm inner diameter and a 100 mm length, and the polymerization was conducted at different polymerization temperatures of −18, 5, 24, and 45 °C for 48 h. Since HyA may decompose at high temperatures, the reactions were performed at 45 °C as the higher polymerization temperature. After the gelation had been completed, the cylindrical gel blocks were removed carefully without destroying their shape and cut into several small samples (Scheme 1). Before the swelling and elasticity measurements, all semi-IPN samples were dialyzed in deionized water to remove by-products and impurities.

### 4.3. Swelling Measurements of HyA-Containing Semi-IPN Gels

A volumetric method was employed to measure the equilibrium volume swelling ratio of semi-IPNs in distilled water at room temperature and buffer solution with different pH values. Semi-IPN samples in cylindrical form were immersed in 100 mL of water or buffer solution after measuring their initial diameter using a calibrated digital compass (Mitutoyo Digimatic Caliper, Series 500, resolution: 0.01 mm). By following the change in diameter of the samples, the measurements were repeated until the equilibrium swelling was reached and no change in the diameters was observed. The equilibrium volume swelling ratio $\varphi_{V}$ was calculated using the following equation:

(7) $$\varphi_{V}=\frac{{\left(D/D_{0}\right)}^{3}}{\nu_{2}^{0}}=\frac{1}{\nu_{2}}$$

where *D* is the diameter of the sample at the equilibrium state. $\nu_{2}^{0}$ and $\nu_{2}$ are the volume fraction of crosslinked polymer in the relaxed state, that is, after preparation-state and at swollen-state, respectively. Using dry weights of the samples, the experimental values of $\nu_{2}^{0}$ were calculated using the following equation:

(8) $$\nu_{2}^{0}={\left[1+\frac{\left(m_{0}/m_{dry}-1\right)\rho_{P}}{d_{w}}\right]}^{-1}$$

where *m_dry_* is the weight of crosslinked sample dried after extraction in water and *m*_0_ is the weight of gel after preparation-state, respectively. *d*_w_ is the density of polymerization solvent, water, 1.0 g/mL, and *ρ*_P_ is the apparent density of the crosslinked network, 1.35 g/mL. The polymer volume ratio, which is an important structural parameter in the characterization of the polymer network, is related to the amount of water that the network can contain. The theoretical values of $\nu_{2}^{0}$ were determined with $\nu_{2,\mathrm{theo}}^{0}=C_{0}{\bar{V}}_{r}/10^{3}$ using the initial molar concentration of the monomers *C*_0_ and the average molar volume of polymer repeating units (in mL/mol) $\bar{V_{r}}$. The gel fraction determined gravimetrically after synthesis and drying was calculated using $w_{gel}\%=(m_{dry}/m_{0})\times 100$.

### 4.4. Mechanical Measurements of HyA-Containing Semi-IPN Gels

The cylindrical gels removed from the syringe were cut in pieces of 5 mm height, and to determine the after-synthesis modulus, the obtained pieces were subjected to uniaxial compression testing using a manual mechanical loading device equipped with a digital comparator (IDC type Digimatic Indicator 543–262, Mitutoyo, Kawasaki, Japan) which is sensitive to displacements of 10^−3^ mm after measuring their diameter and weight. For the determination of the swelling state modulus, the gels first reached the swelling equilibrium in water and were compressed after measuring their swollen diameter and weight. Four samples of each composition were used, and the measurements were performed using a crosshead speed of 5 mm min^−1^. In all measurements, the test parameters were set as follows: the compression was completed when 10% of the sample length was deformed, the relaxation time of the polymer chains was set to 10 s, and each test was completed in less than 3 min to prevent the water loss during the measurement. The compressive elastic modulus, *G*, was determined from the slope of linear dependence described using the simplified Mooney–Rivlin equation [51,52,53], as follows:

(9) $$\sigma =f/S=G(\alpha -\alpha^{-2})$$

where σ is the nominal stress in Pa m^−2^, *f* is the value of measured force, *S* is the cross-section of the undeformed sample, $S=\pi {\left(D_{0}/2\right)}^{2}$, and α is the relative deformation ratio of the sample as $\alpha =1-\Delta L/L_{0},$ in which Δ*L* is the displacement, the change in length of the compressed sample, and determined by the initial *L*_0_ and compressed sample length *L* along the single axis as $\Delta L=L_{0}-L.$

### 4.5. Attenuated Total Reflectance Fourier Transform Infrared Spectroscopy (ATR-FTIR)

The prepared semi-IPN gels were studied using a Perkin Elmer Spectrum 100 FTIR in the attenuated total reflectance mode (ATR-FTIR). Before the FTIR measurements, the gels in the fully dried state were finely ground. The scans were recorded using a constant universal compression force at room temperature in the wavenumber range of 4000–650 cm^−1^ with a resolution of 4 cm^−1^. X-ray diffraction (XRD) analysis was performed by Philips PW-3710 for structural analyses. XRD measurements were conducted with a copper emission source (CuKα = 1.5418 Ǻ) in the 2*θ* range from 4° to 50° by 0.02°/0.5 s scanning rate operated at 40 KeV and current 25 mA.

### 4.6. Adsorption Efficiency of HyA-Interpenetrated Semi-IPNs for Cationic Dyes

To study the adsorption capacity of semi-IPNs with varying HyA loading, cationic dye methyl violet (MV) was used as a model dye. 0.010 g of dry semi-IPN sample was added to 15 mL of 20 mg/L MV dye solution under continuous stirring using an orbital shaker at 100 rpm at ambient temperature. After reaching equilibrium adsorption capacity, the solutions were filtered using centrifugation, and the remaining dye content was measured using a UV–Vis spectrophotometer (Novel N4S/N4 UV-Visible Spectrophotometer) at 577 nm. Using the calibration plot of MV (A = 0.01793 + 0.1162C, R^2^ = 0.9987), the adsorption capacity of the adsorbents at equilibrium (*q_e_*, mg L^−1^) and adsorption efficiency were calculated using the following equations:

(10) $$q_{e}=\frac{(C_{i}-C_{e})V}{m}$$

(11) $$Adsorption\;(\%)=\frac{\left(C_{i}-C_{t}\right)}{C_{i}}\times 100$$

where *C_i_* is the initial concentration of MV dye, *C_t_* is the final concentration, *V* is the volume of dye solution used, and *m* (g) is the mass of adsorbent used. Four kinetic models, pseudo-first-order (PFO), pseudo-second-order (PSO), Avrami, fractional, Elovich, and intraparticle diffusion models, were employed in kinetic data analyses in linear and nonlinear forms.

## Figures and Tables

**Table 1:** Synthesis route and network composition of PAAm/PSA/HyA semi-IPNs containing various amounts of HyA formed at different preparation temperatures (Tprep, °C) and experimental values of the volume fraction of crosslinked polymer network after preparation.

**AAm content**	710.8 mg			$\nu_{2}^{0}$ **(Experimental)**			
**PSA content**	150.0 mg	**Sample** **Designation**	**HyA %** **(*w*/*v*)**	**Tprep (°C)**			
**HyA content**	0.05–0.20% (*w*/*v*)			**−18**	**5**	**24**	**45**
**Crosslinking agent BAAm**	19.4 mg	**PAAm/PSA/HyA0**	0	0.0493	0.0573	0.0546	0.0595
**APS conc**	3.51 mM	**PAAm/PSA/HyA1**	0.05	0.0546	0.0612	0.0554	0.0601
**TEMED conc.**	24.9 mM	**PAAm/PSA/HyA2**	0.10	0.0610	0.0662	0.0582	0.0721
**Polymerization temperature**	−18, 5, 24, 45 °C	**PAAm/PSA/HyA3**	0.15	0.0574	0.0633	0.0606	0.0608
		**PAAm/PSA/HyA4**	0.20	0.0547	0.0632	0.0646	0.0606

**Table 2:** Parametric values of linearized PFO, PSO, Elovich, Avrami, fractional, and intraparticle diffusion kinetic model constants determined from the experimental data of 0.2% (*w*/*v*) HyA-containing PAAm/PSA/HyA4 gels.

**Pseudo-first-order model (PFO)**				**Elovich model**			
**Tprep/°C**	$k_{1}\times 10^{-2}$ **(min^−1^)**	**R^2^**			α **(mg/g min)**	β **(g/mg)**	**R^2^**
**45**	1.8992	0.9081			77.741	0.1352	0.8666
**24**	1.3441	0.9657			5.6575	0.1063	0.9786
**5**	1.4048	0.8534			11.831	0.1362	0.9867
**−18**	1.7457	0.9139			11.526	0.1151	0.9761
**Pseudo-second-order model (PSO)**				**Intraparticle model**			
**Tprep/°C**	$k_{2}\times 10^{-3}$ **(g/mg min^−1^)**	**R^2^**		$k_{initial}$ **(mg g^−1^ min^−1/2^)**	**R^2^**	$k_{later}$ **(mg g^−1^ min^−1/2^)**	**R^2^**
**45**	1.4611	0.9999		4.1629	0.8963	0.3424	0.9496
**24**	0.5731	0.9992		3.7782	0.9908	0.8630	0.9854
**5**	0.7731	0.9983		2.7207	0.9742	0.7668	0.9777
**−18**	0.7305	0.9992		3.4728	0.9589	0.7487	0.9355
**Fractional power model**				**Avrami model**			
**Tprep/°C**	$\nu_{fr}$ **(min^−1^)**	$k_{fr}$ **(mg/g)**	**R^2^**		$n_{\mathrm{Av}}$	$k_{\mathrm{Av}}\times 10^{-2}$ **(min^−1^)**	**R^2^**
**45**	0.4294	212.19	0.9735		0.4071	5.0813	0.9317
**24**	0.4227	71.537	0.9889		0.5611	1.7451	0.9923
**5**	0.2843	563.98	0.9844		0.3918	2.7510	0.9829
**−18**	0.3460	650.61	0.9751		0.5269	2.4790	0.9925

**Table 3:** Thermodynamic parameters for the adsorption of MV and adsorption capacity of 0.2% (*w*/*v*) HyA-containing PAAm/PSA/HyA4 gels.

		Linearized Model		Non-Linearized Model		
Tprep/°C	Exp. $q_{e}$ (mg/g)	PFO Model $q_{e}$ (mg/g)	PSO Model $q_{e}$ (mg/g)	PFO Model $q_{e}$ (mg/g)	PSO Model $q_{e}$ (mg/g)	$\Delta G^{0}$ (kJ/mol K)
**45**	61.264	38.983	59.892	56.797	60.476	−8.133
**24**	52.715	42.914	53.545	48.392	52.929	−7.556
**5**	50.834	38.931	49.173	44.718	48.558	−7.165
**−18**	54.476	43.118	55.479	50.279	54.345	−7.081

## Data Availability

The original contributions presented in this study are included in the article/Supplementary Materials; further inquiries can be directed to the corresponding author/s.

## Supplementary Materials

The following supporting information can be downloaded at https://www.mdpi.com/article/10.3390/gels10090556/s1. Figure S1: (A) The crosslink density νe of PAAm/PSA/HyA gels formed at 5, 24 and 45 °C plotted against HyA %. (B) Uniaxial compression of PAAm/PSA/HyA4 gels containing 0.2% (*w*/*v*) formed at 5, and 24 °C; Figure S2: Optical images of HyA-integrated PAAm/PSA/HyA gels after 1 h adsorption of MV dye; Figure S3: The adsorption performance of HyA-integrated PAAm/PSA/HyA gels with varying HyA content as a function of contact time at different preparation temperatures; Tprep (oC) = 45 (A), 24 (B), 5 (C) and -18 (D); Figure S4: Regression analysis of adsorption of MV with 0.1% (*w*/*v*) HyA-integrated PAAm/PSA/HyA2 gels by pseudo-first-order (PFO) kinetic model (A), pseudo-second order (PSO) kinetic model (B), Avrami model (C) and intra-particle diffusion model (D); Figure S5: Regression analysis of adsorption of MV onto 0.10% (*w*/*v*) (A, C) and 0.2% (*w*/*v*) (B, D) HyA-integrated PAAm/PSA/HyA gels by linearized Elovich (A, B) and fractional (C, D) kinetic model; Figure S6: Results of non-linear PFO kinetic model, PSO kinetic model, Avrami, Elovich kinetic model, and intraparticle diffusion model of 0.10% (*w*/*v*) PAAm/PSA/HyA2 semi-IPN gels formed at polymerization temperature of Tprep (oC): 45 (A), 24 (B), 5 (C) and -18 (D); Figure S7: Optical images of 0.1% (*w*/*v*) HyA-integrated PAAm/PSA/HyA2 gels after 1 h adsorption of MV dye. Table S1. Attribution of main bands in FTIR spectra of PAAm/PSA and semi-IPN PAAm/PSA/HyA gels; Table S2. The equations used for pseudo-first-order, pseudo-second-order, Elovich, Avrami kinetic, and intra-particle model for total MV adsorption onto hybrid gels; Table S3. The comparison of linearized PFO, PSO, Elovich, Avrami, fractional and intraparticle diffusion kinetic models’ rate constants calculated from the experimental adsorption data of 0.1% (*w*/*v*) HyA-containing PAAm/PSA/HyA2 gels; Table S4. The parametric values of non-linearized PFO, PSO, Elovich, and Avrami kinetic models’ rate constants calculated from the experimental adsorption data of 0.2% (*w*/*v*) HyA-containing PAAm/PSA/HyA4 gels; Table S5. The parametric values of non-linearized PFO, PSO, Elovich, and Avrami kinetic models’ rate constants calculated from the experimental adsorption data of 0.1% (*w*/*v*) HyA-containing PAAm/PSA/HyA2 gels; Table S6. Thermodynamic parameters for the adsorption of MV and adsorption capacity of 0.1% (*w*/*v*) HyA-containing PAAm/PSA/HyA2 gels.
