# Supplementary material for: Temperature-Regulated Synthesis of Hyaluronic Acid-Interpenetrated Polyacrylamide/Poly(Acrylic Acid Sodium Salt) Semi-Interpenetrated Polymer Network Gel for the Removal of Methyl Violet
Source: Gels. 2024 Aug 28;10(9):556. doi: 10.3390/gels10090556 (PMC11431609; doi:10.3390/gels10090556)
Supplement: Supplementary file 1 [file gels-10-00556-s001.zip › gels-3162384-supplementary.pdf]

Temperature regulated synthesis of hyaluronic acid-interpenetrated polyacrylamide/poly(acrylic acid sodium salt) semi-IPN gel for removal of methyl violet

Nida ÖZCAN[a] and Nermin ORAKDOGEN[b]*

[a]Graduate School of Science Engineering and Technology, Department of Chemistry, Istanbul Technical University, 34469, Maslak, Istanbul, Turkey

[b]Istanbul Technical University, Faculty of Science and Letters, Department of Chemistry, Soft Materials Research Laboratory, 34469, Maslak, Istanbul, Turkey, Tel: +90-212-285-3305, Fax: +90-212-285-6386
E-mail: orakdogen@itu.edu.tr

Structural characterization of HyA-integrated semi-IPN gels

Table S1. Attribution of main bands in FTIR spectra of PAAm/PSA and semi-IPN PAAm/PSA/HyA gels.

Sample			Characteristic Functional Group Assignments
HyA	PAAm/PSA	PAAm/PSA/HyA	
-	3405	3410	νas NH2 of AAm units
-	3190	3196	νs NH2 of AAm units
-	1579	1579	νas COO- of NaA
2929,2860	2929,2857	2931,2850	νCH and νCH2
	1640	1649	carbonyl C=O stretching vibration (amide I)

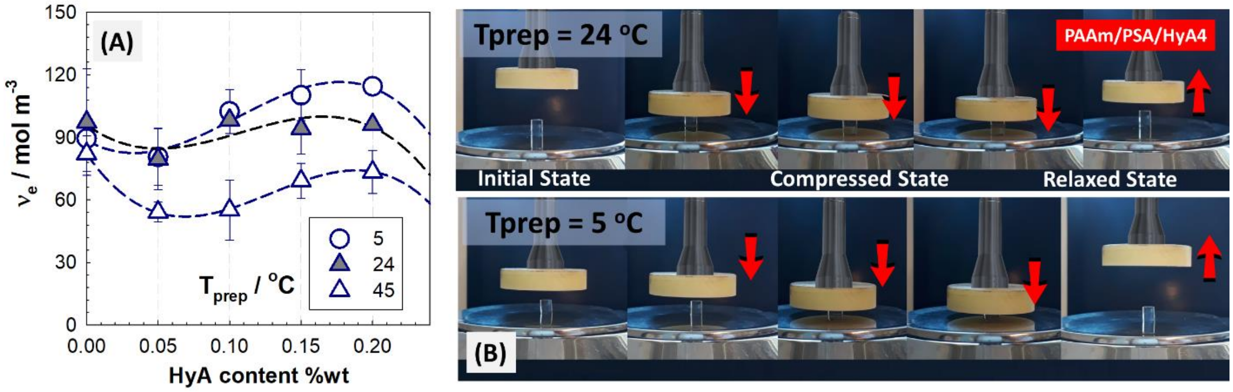

Figure S1. (A) The cross-link density νe of PAAm/PSA/HyA gels formed at 5, 24 and 45 °C plotted against HyA %. (B) Uniaxial compression of PAAm/PSA/HyA4 gels containing 0.2% (w/v) formed at 5, and 24 °C.

Adsorption properties of HyA-loaded PAAm/PSA/HyA gels

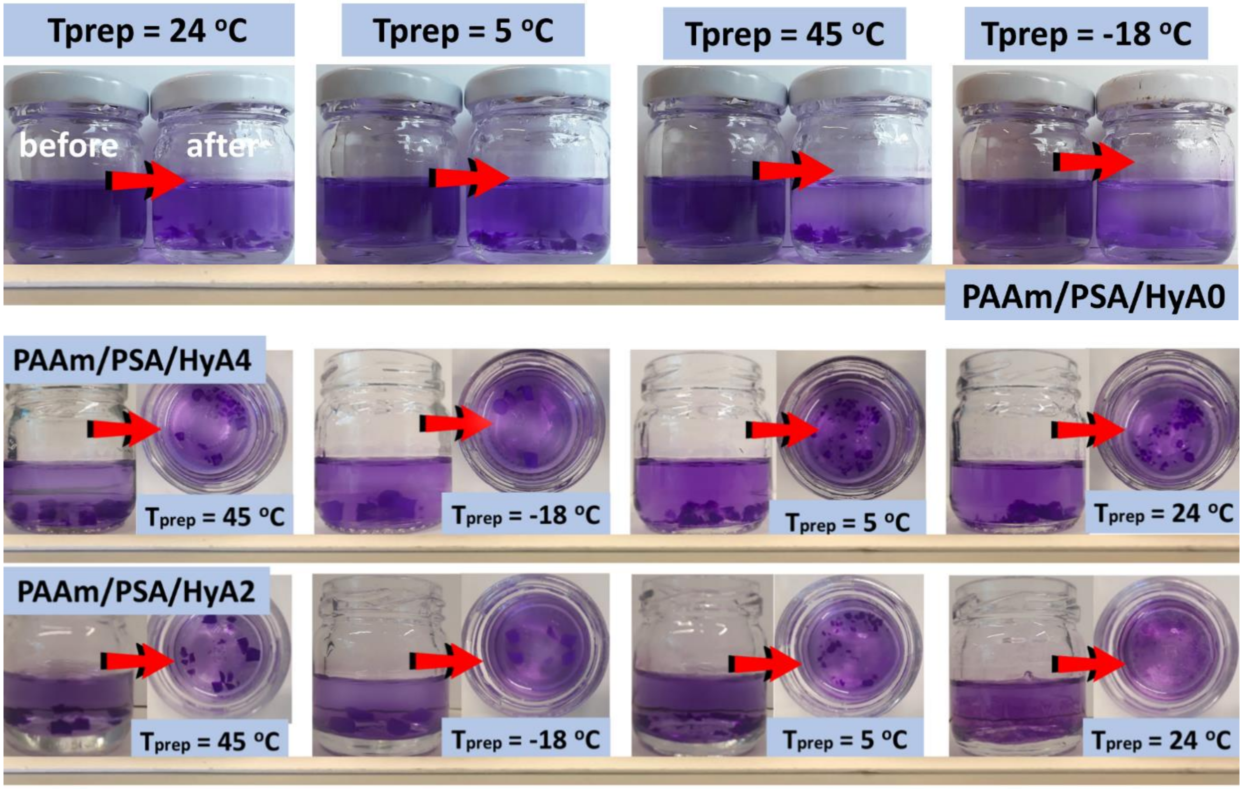

Figure S2. Optical images of HyA-integrated PAAm/PSA/HyA gels after 1 h adsorption of MV dye.

Table S2. The equations used for pseudo-first-order, pseudo-second-order, Elovich, Avrami kinetic, and intra-particle model for total MV adsorption onto hybrid gels.

	Adsorption Kinetic Model	Linearized Equation	Non-linearized Equation	Kinetic parameters
**Eq.(S1)**	Pseudo-First-order	$\ln(q_e - q_t) = \ln q_e - k_1 t$	$q_t = q_e(1 - e^{-k_1 t})$	k_1 is pseudo-first-order rate constant (min^{-1})
**Eq.(S2)**	Pseudo-Second-order	$\frac{t}{q_t} = \frac{t}{q_e} + \frac{1}{k_2 q_e^2}$	$q_t = \frac{k_2 q_e^2 t}{1 + k_2 q_e t}$	k_2 is pseudo-second-order rate constant ($g\ mg^{-1}\ min^{-1}$)
**Eq.(S3)**	Elovich	$q_t = \frac{1}{\beta}\ln t + \frac{1}{\beta}\ln(\alpha\beta)$	$q_t = \frac{1}{\beta}\ln(\alpha\beta t)$	α is a constant for rate of chemisorption, β is a constant for extent of surface coverage of adsorbent
**Eq.(S4)**	Avrami	$\ln\left[\ln\left(\frac{q_e}{q_e - q_t}\right)\right] = n_{Av}\ln k_{Av} + n_{Av}]$	$q_t = q_e\left[1 - e^{\left[-(k_{Av}\ t)^{n_{Av}}\right]}\right]$	k_{Av} is Avrami kinetic constant, and n_{Av} is Avrami exponent
**Eq.(S5)**	Fractional power	$\ln q_t = \ln k_{fr} + \nu_{fr}\ln t$	$q_t = k_{fr} t^{n_{fr}}$	k_{fr} and ν_{fr} are the fractional kinetic constants
**Eq.(S6)**	Intraparticle diffusion model	$q_t = k_{diff} t^{1/2} + C$	$q_t = k_{diff} t^{1/2}$	k_{diff} is rate constant for intraparticle diffusion ($mg\ g^{-1}\ min^{-1/2}$), and C is a constant for thickness of boundary layer.

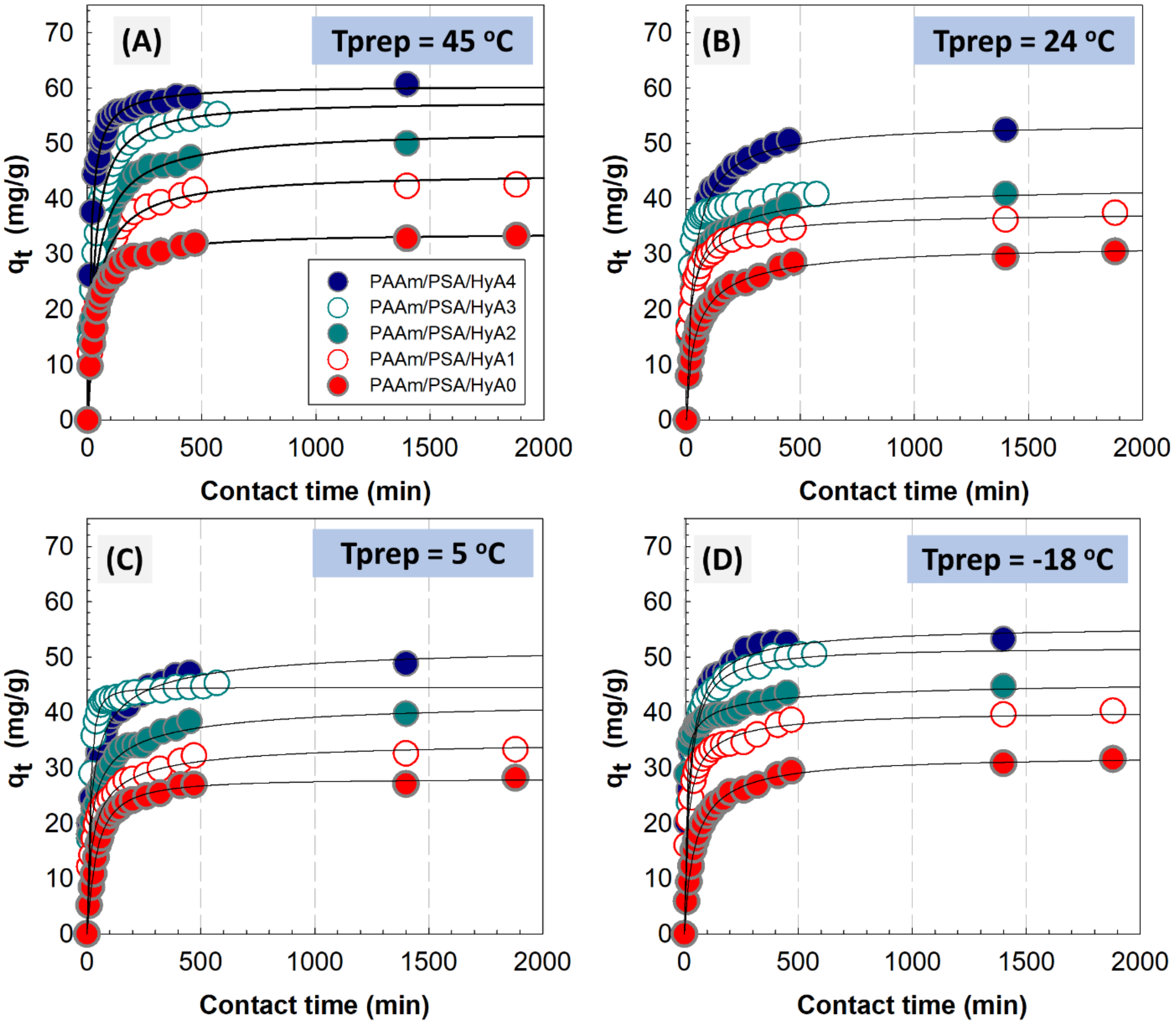

Figure S3. The adsorption performance of HyA-integrated PAAm/PSA/HyA gels with varying HyA content as a function of contact time at different preparation temperatures; Tprep (ºC) = 45 (A), 24 (B), 5 (C) and -18 (D).

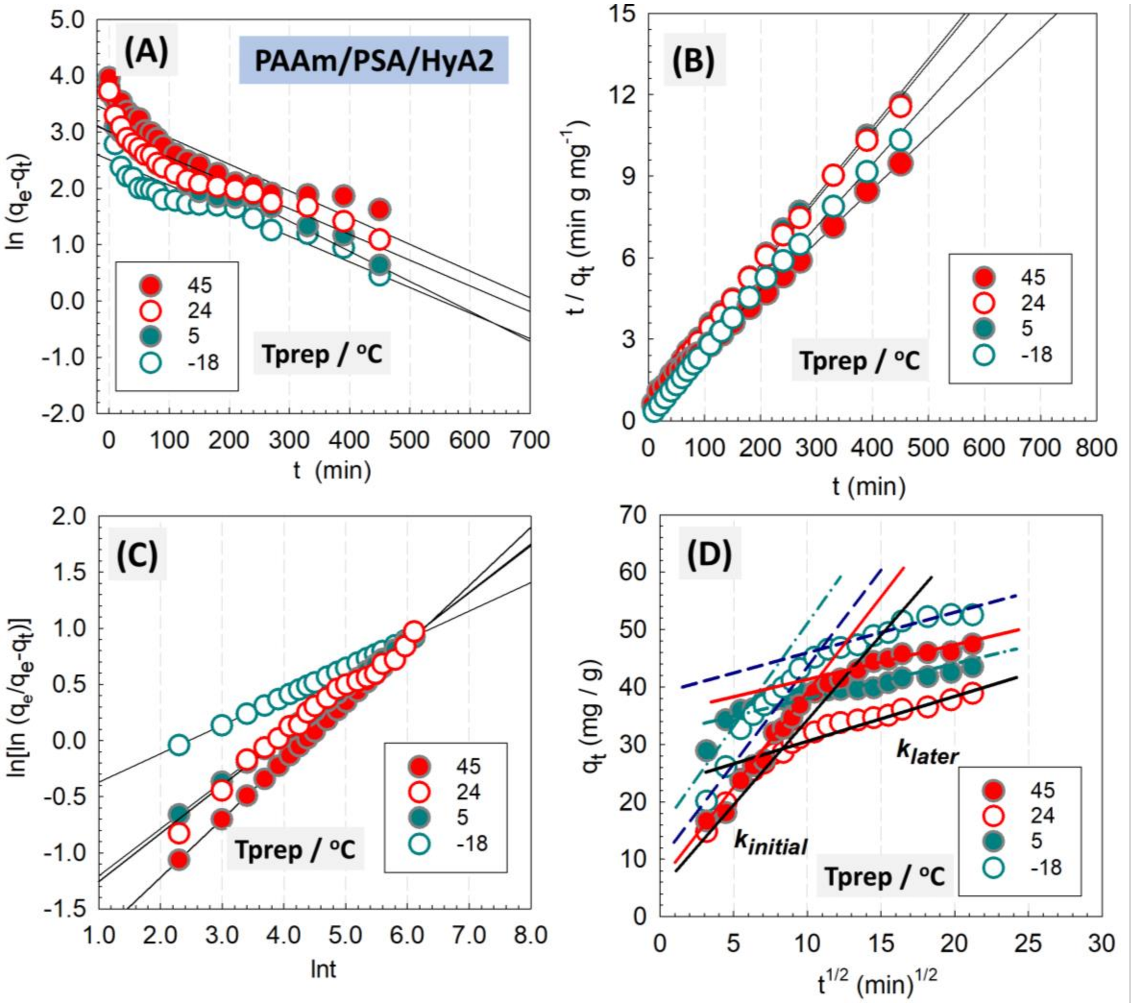

Figure S4. Regression analysis of adsorption of MV with 0.1% (w/v) HyA-integrated PAAm/PSA/HyA2 gels by pseudo-first-order (PFO) kinetic model (A), pseudo-second order (PSO) kinetic model (B), Avrami model (C) and intra-particle diffusion model (D).

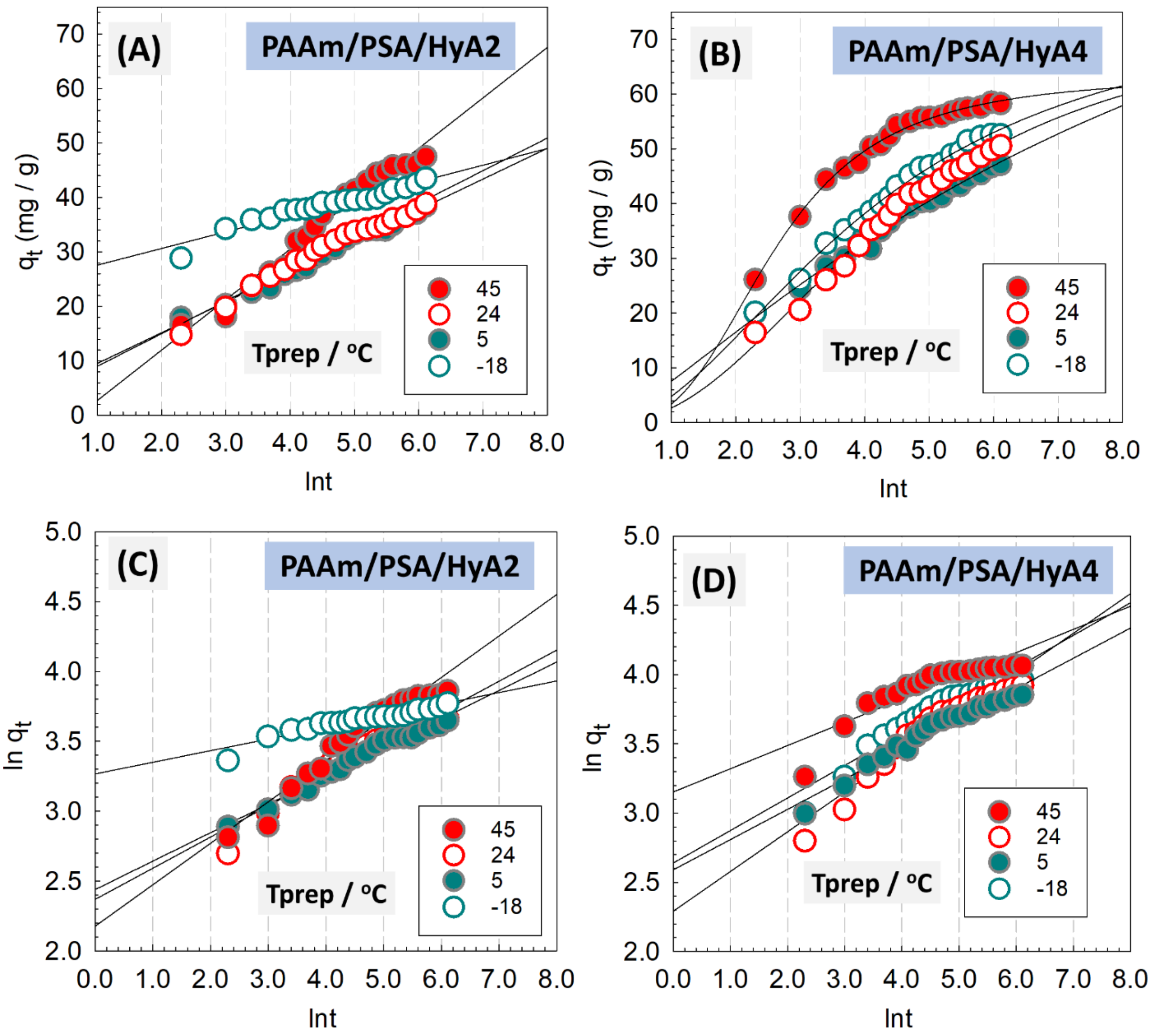

Figure S5. Regression analysis of adsorption of MV onto 0.10% (w/v) (A, C) and 0.2% (w/v) (B, D) HyA-integrated PAAm/PSA/HyA gels by linearized Elovich (A, B) and fractional (C, D) kinetic model.

Table S3. The comparison of linearized PFO, PSO, Elovich, Avrami, fractional and intraparticle diffusion kinetic models' rate constants calculated from the experimental adsorption data of 0.1% (w/v) HyA-containing PAAm/PSA/HyA2 gels.

Pseudo-first order model (PFO)			Elovich model		
Tprep / °C	$k_1 \times 10^{-2}$ (min^{-1})	R^2	α (mg/g min)	β (g/mg)	R^2
45	0.4740	0.8539	4.5840	0.1080	0.9672
24	0.4569	0.8625	9.9609	0.1671	0.9779
5	0.5330	0.9157	11.295	0.1773	0.9864
-18	0.4540	0.7417	7.0061	0.3271	0.9295

Pseudo-second order model (PSO)			Intra-particle model			
Tprep / °C	$k_2 \times 10^{-3}$ (g / mg min^{-1})	R^2	$k_{initial}$ (mg g^{-1} min$^{-1/2}$)	R^2	k_{later} (mg g^{-1} min$^{-1/2}$)	R^2
45	0.5719	0.9986	3.1557	0.9820	0.6514	0.8939
24	1.0157	0.9983	2.4429	0.9579	0.5728	0.9834
5	0.9371	0.9972	1.5860	0.8403	0.3906	0.9554
-18	2.2719	0.9986	3.5927	0.9550	0.8131	0.9347

	Fractional power model			Avrami model		
Tprep / °C	v_{fr} (min^{-1})	k_{fr} (mg / g)	R^2	n_{Av}	$k_{Av} \times 10^{-2}$ (min^{-1})	R^2
45	0.3823	129.55	0.9747	0.5264	1.3636	0.9766
24	0.3626	183.03	0.9842	0.4294	1.9730	0.9840
5	0.2201	504.57	0.9865	0.4279	2.1824	0.9816
-18	0.2470	895.17	0.8982	0.2454	0.9462	0.9397

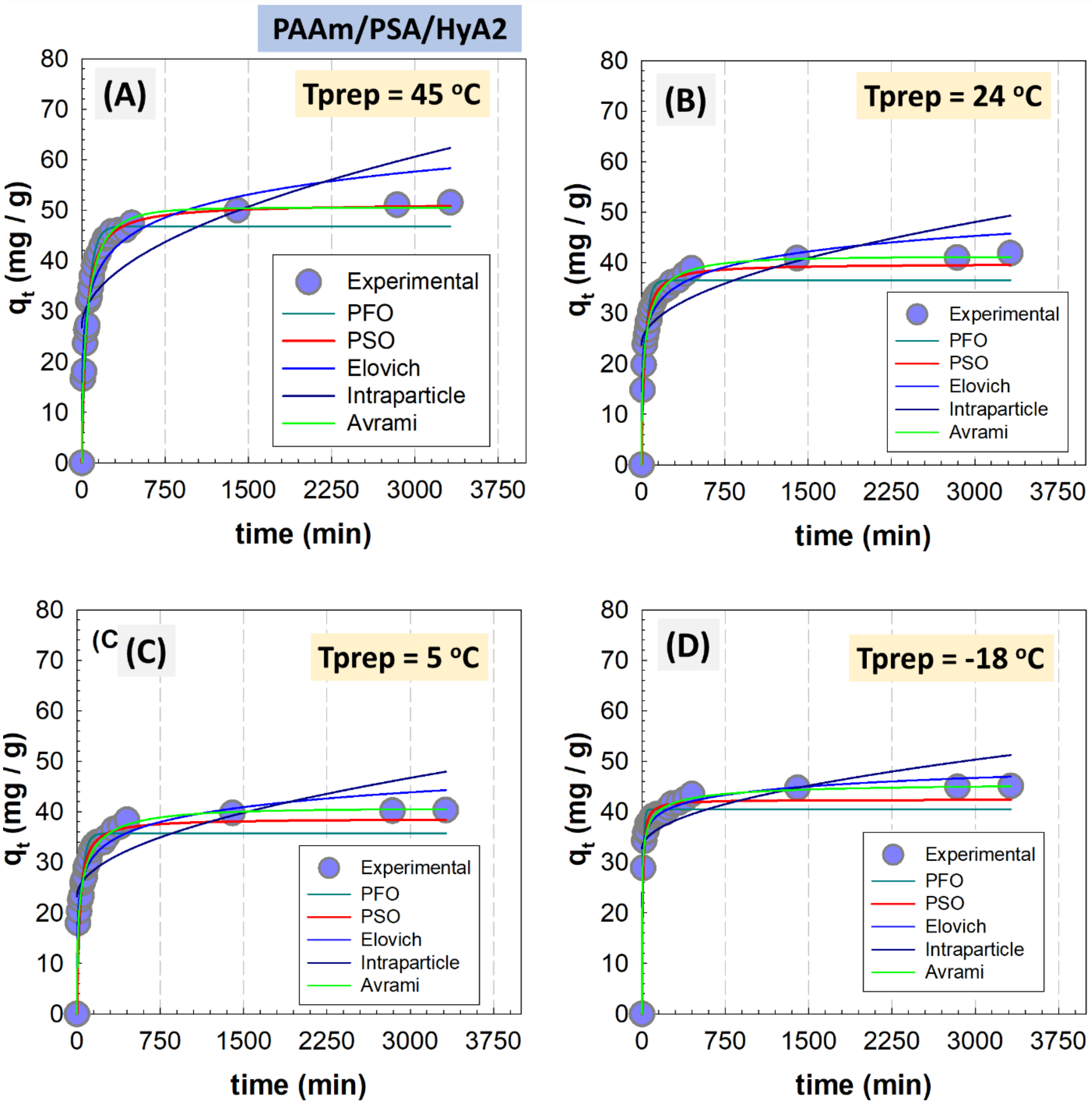

Figure S6. Results of non-linear PFO kinetic model, PSO kinetic model, Avrami, Elovich kinetic model, and intraparticle diffusion model of 0.10% (w/v) PAAm/PSA/HyA2 semi-IPN gels formed at polymerization temperature of Tprep (°C): 45 (A), 24 (B), 5 (C) and -18 (D).

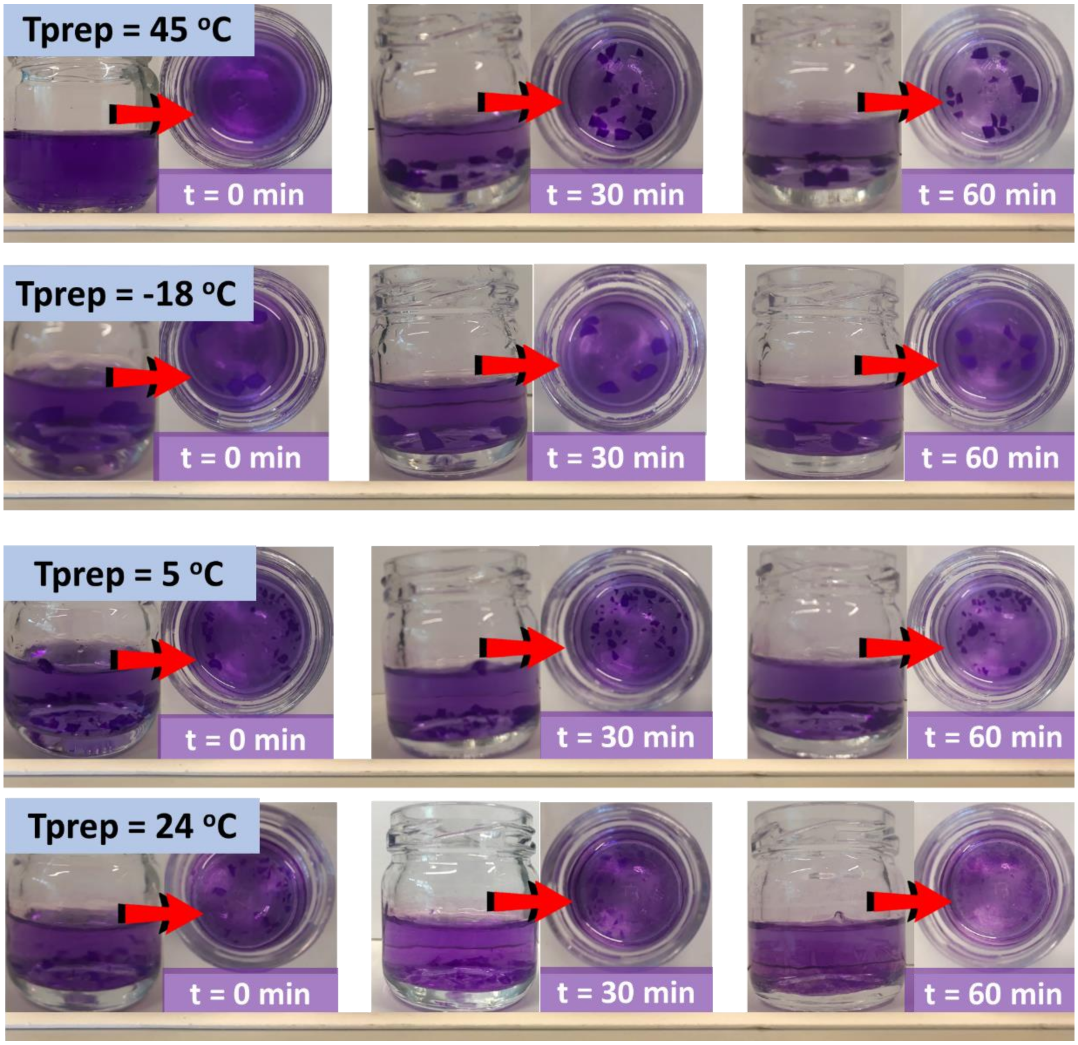

Figure S7. Optical images of 0.1% (w/v) HyA-integrated PAAm/PSA/HyA2 gels after 1 h adsorption of MV dye.

Table S4. The parametric values of non-linearized PFO, PSO, Elovich, and Avrami kinetic models' rate constants calculated from the experimental adsorption data of 0.2% (w/v) HyA-containing PAAm/PSA/HyA4 gels.

Pseudo-first order model (PFO)			Elovich model		
Tprep / °C	$k_1 \times 10^{-2}$ (min^{-1})	R^2	α (mg/g min)	β (g/mg)	R^2
**45**	4.7556	0.9646	44.419	0.2084	0.9032
**24**	2.1750	0.9530	21.946	0.1549	0.9109
**5**	2.7517	0.8922	48.153	0.1851	0.9513
**-18**	2.8636	0.9385	78.322	0.1737	0.9113

Pseudo-second order model (PSO)			Avrami model		
Tprep / °C	$k_2 \times 10^{-3}$ (g / mg min^{-1})	R^2	n_{Av}	$k_{Av} \times 10^{-2}$ (min^{-1})	R^2
**45**	1.3500	0.9975	0.5839	4.908	0.9928
**24**	0.6110	0.9950	0.5870	1.845	0.9969
**5**	0.8853	0.9759	0.4448	2.124	0.9967
**-18**	0.8421	0.9931	0.5399	2.585	0.9977

Table S5. The parametric values of non-linearized PFO, PSO, Elovich, and Avrami kinetic models' rate constants calculated from the experimental adsorption data of 0.1% (w/v) HyA-containing PAAm/PSA/HyA2 gels.

Pseudo-first order model (PFO)			Elovich model		
Tprep / °C	$k_1 \times 10^{-2}$ (min^{-1})	R^2	α (mg/g min)	β (g/mg)	R^2
**45**	1.891	0.9493	14.060	0.1521	0.9170
**24**	2.881	0.9072	38.530	0.2244	0.9509
**5**	2.774	0.8628	44.487	0.2362	0.9572
**-18**	10.36	0.9231	17.362	0.4106	0.9843

Pseudo-second order model (PSO)			Avrami model		
Tprep / °C	$k_2 \times 10^{-3}$ (g / mg min^{-1})	R^2	n_{Av}	$k_{Av} \times 10^{-2}$ (min^{-1})	R^2
**45**	0.5412	0.9886	0.5920	1.587	0.9932
**24**	1.1124	0.9838	0.4476	2.169	0.9955
**5**	1.1550	0.9603	0.4148	2.089	0.9954
**-18**	4.1805	0.9751	0.2363	16.07	0.9947

Table S6. Thermodynamic parameters for the adsorption of MV and adsorption capacity of 0.1% (w/v) HyA-containing PAAm/PSA/HyA2 gels.

		Linearized model		Non-linearized model		
**Tprep / °C**	Exp. q_e (mg/g)	PFO model q_e (mg/g)	PSO model q_e (mg/g)	PFO model q_e (mg/g)	PSO model q_e (mg/g)	ΔG^o (kJ/mol K)
**45**	52.565	29.304	50.902	46.806	51.348	-8.7443
**24**	41.895	20.307	39.860	36.555	39.803	-8.0608
**5**	40.344	20.389	39.492	35.725	38.696	-7.4813
**-18**	45.122	12.319	43.393	40.471	42.420	-7.2512
